# Influencing the Insulin System by Placebo Effects in Patients With Diabetes Type 2 and Healthy Controls: A Randomized Controlled Trial

**DOI:** 10.1097/PSY.0000000000001216

**Published:** 2023-06-23

**Authors:** Aleksandrina Skvortsova, Dieuwke S. Veldhuijzen, Lotte F. van Dillen, Hilmar Zech, Suzanne M.J.C. Derksen, Ruben H. Sars, Onno C. Meijer, Hanno Pijl, Andrea W.M. Evers

**Affiliations:** From the Department of Psychology (Skvortsova), McGill University, Montreal, Quebec, Canada; Health, Medical and Neuropsychology Unit (Skvortsova, Veldhuijzen, Zech, Derksen, Sars, Evers), and Social, Economic and Organizational Psychology Unit (van Dillen), Faculty of Social and Behavioural Sciences, Leiden University, Leiden, the Netherlands; Neuroimaging Center (Zech), Dresden University of Technology, Dresden, Germany; Department of Medicine, Division of Endocrinology (Meijer, Pijl), Leiden University Medical Centre; Department of Psychiatry (Evers), Leiden University Medical Center, Leiden; and Medical Delta, Leiden University (Evers), Technical University Delft and Erasmus University, Delft, the Netherlands.

**Keywords:** pharmacological conditioning, placebo effect, intranasal insulin, type 2 diabetes, glucose, **CS** = conditioned stimulus, **US** = unconditioned stimulus

## Abstract

**Objective:**

The objective of this study was to investigate whether placebo effect induced by pharmacological conditioning with intranasal insulin can affect glucose, insulin, C-peptide, hunger, and memory in patients with diabetes type 2 and healthy controls.

**Methods:**

Placebo effect was induced by pharmacological conditioning. Thirty-two older patients (mean age = 68.3 years) with diabetes type 2 and age- and sex-matched thirty-two healthy older adults (mean age = 67.8 years) were randomly assigned to a conditioned or a control group. On day 1, conditioned group received six administrations of intranasal insulin with a conditioned stimulus (CS; smell of rosewood oil), whereas the control group received a placebo with the CS. On day 2, both groups received a placebo spray with the CS. Glucose, insulin, and C-peptide were repeatedly measured in blood. Hunger and memory were assessed with validated measures.

**Results:**

Intranasal insulin stabilized dropping glucose levels in patients (*B* = 0.03, SE = 0.02, *p* = .027) and healthy men (*B* = 0.046, SE = 0.02, *p* = .021), and decreased C-peptide levels in healthy controls (*B* = 0.01, SE = 0.001, *p* = .008). Conditioning also prevented the drop of glucose levels but only in men (both healthy and patients; *B* = 0.001, SE = 0.0003, *p* = .024). Conditioning significantly decreased hunger in healthy participants (*B* = 0.31, SE = 0.09, *p* < .001). No effects were found on other measures.

**Conclusions:**

Placebo effect induced by conditioning with intranasal insulin modifies blood glucose levels and decreases hunger in older adults, but its effects depend on health status and sex. Insulin conditioning might be beneficial for groups suffering from intensive hunger but seems not be particularly suitable for blood glucose reduction.

**Trial Registration:**

Netherlands Trial Register, NL7783 (https://www.trialregister.nl/trial/7783).

## INTRODUCTION

Placebo effects are positive treatment outcomes that cannot be attributed to the pharmacological mechanisms of the treatment but are caused by the psychosocial context (1). Placebo effects can be induced by positive patient-doctor communication, observational learning, or associative leaning through classical conditioning procedures. Accumulating evidence suggests that it is possible to modulate endocrine functions using classical conditioning (2,3): coupling of an active medication (unconditioned stimulus [US]) with an initially neutral stimulus (conditioned stimulus [CS]). In case of endocrine conditioning, hormonal-stimulating or inhibiting medication (US) gets associated with the CS, and later, the mere presentation of the CS alone leads to changes in hormone levels or triggers effects associated with this hormone.

Several possible clinical applications of placebo effects induced by conditioning were proposed (4,5). For example, dosages of standard treatments can be reduced using placebo-controlled dose reduction protocols, in which an active drug gets pharmacologically conditioned and then a part of it is replaced by a placebo while maintaining the efficacy of treatment (4). Placebo effects can also boost the efficiency of treatments when conditioning procedures are added to the standard treatment protocols (6).

The most convincing evidence for endocrine conditioning comes from studies on conditioning of insulin and glucose responses in animals and healthy humans (7–11). Insulin and glucose responses seem to be particularly malleable by the mechanisms of conditioning (12), probably because of their acute homeostatic functions aimed at maintaining glucose metabolism. Cephalic phase release of insulin, for example, is a transient pulse of insulin that has been observed in both animals and humans in response to food cues, such as the smell of food, or the time of the day when food is regularly taken (12). This conditioned response seems to help prepare the organism for the upcoming homeostatic changes related to the food consumption and prevent hyperglycemia caused by consumption of large amounts of food (13). Not only naturally occurring associations, such as associations between the smell of food and food intake, can trigger conditioned insulin responses. Experimental studies demonstrated that coupling of food with any neutral stimuli, such as a sound or a light, can trigger conditioned insulin release (14–16). Moreover, insulin and glucose responses can be conditioned using US other than food. Using insulin injections as a US, it was found possible to classically condition glucose decrease in healthy young volunteers (9,10). Another study successfully conditioned insulin release and glucose decrease in healthy volunteers using intranasal insulin administration as a US (7).

Up-to-date, most of the animal research on insulin conditioning has been done in male mice or rats (for the review, see Ref. (2)), and the few available human studies were performed in young male volunteers. Therefore, it remains unknown whether sex or age might play any role in the conditionability of insulin effects. Importantly, there are no reports of the possibility to condition insulin responses in metabolic disorders. Particularly, patients with diabetes type 2 might benefit from conditioning with intranasal insulin as an US because intranasal insulin has been shown to have a number of benefits for patients with diabetes type 2. Conditioning with insulin might trigger conditioned insulin release and glucose decrease (7) without causing common adverse effects of intravenous insulin injections such as hypoglycemia and hypertension (17). Moreover, because intranasal insulin normalizes hypothalamic neuronal activity in response to glucose ingestion, it could be especially favorable for type 2 diabetes patients who demonstrate distorted brain responses to glucose (18,19). In addition, evidence suggests that intranasal insulin decreases food intake and hunger (20,21) and improves memory both in healthy volunteers and patients with diabetes type 2 (22,23). Taken together, classical conditioning with intranasal insulin has a wide range of potential positive effects for patients with diabetes type 2.

The aim of the present study was to investigate the effects of conditioning with intranasal insulin on blood glucose, insulin, C-peptide, hunger, and memory in a group of patients with diabetes type 2 and age- and sex-matched healthy controls. In addition, we aimed to explore differences between healthy individuals and patients with diabetes type 2, as their responses to insulin and conditioning might differ because of insulin resistance (24) or different baseline levels of glucose (25) or metabolic hormones (26,27). Finally, we explored possible sex differences in the effects of conditioning with intranasal insulin.

## METHODS

### Participants

Patients diagnosed with diabetes type 2 and healthy controls were included in the study. Healthy controls were matched for age (the mean age of the groups was matched ±1 year) and sex to the patients’ group. Inclusion criteria for the patients were as follows: a) being older than 18 years, b) current diagnosis of diabetes type 2, and c) taking metformin and/or participating in a lifestyle intervention (e.g., diet) to control their diabetes. Exclusion criteria for both healthy subjects and patients were as follows: a) use of insulin or insulin stimulating medications; b) use of medication that influences glucose metabolism (e.g., corticosteroid medication, chemotherapy, β-blockers); c) diagnosis of a chronic noncommunicable disease (degenerative diseases, malignant neoplasms such as cancer, diabetes type 1, autoimmune diseases); d) diagnosis of an acute infectious disease (such as meningitis, hepatitis B, and bacterial pneumonia); e) current diagnosis of a mental disorder; f) chronic and/or acute rhinitis; g) anatomic deviations of the nose; h) substance abuse (e.g., drugs or alcohol); and i) pregnancy.

The sample size calculation was based on the results of the study with a comparable design in healthy participants, which reported an effect size of *d* = 0.77 (7). A power analysis using this effect size yielded that 16 participants per condition and per group are needed with a power of 0.8 and a two-sided *α* of .05 as determined by G*Power software.

### Study Design

The study had a double-blind, randomized, placebo-controlled design. Thirty-two patients with diabetes type 2 and 32 healthy controls were randomized to one of two groups in a double-blind manner: a) conditioned group and b) control group. Men and women were equally distributed between the groups. This study was an adaptation of the study design used by Stockhorst and colleagues (7) for conditioning insulin responses in healthy participants. The study conditions are presented in Figure S1, Supplemental Digital Content, http://links.lww.com/PSYMED/A936.

The study was approved by the Medical Ethical Committee of Leiden, Den Haag, Delft, under protocol number P18.222.

Randomization was performed by the Department of Clinical Pharmacy of the Leiden University Medical Center. A block randomization was used with a size of eight participants per block. Equal numbers of men and women were randomized to each condition. The pharmacy was responsible for assigning participants to the conditions. The experimenter was blinded regarding the conditions, and the list coupling participants numbers with conditions remained at the pharmacy until the last participant was tested.

### Procedure

The data collection was done from May 2019 to March 2021. The study procedures are presented in Figure S2, Supplemental Digital Content, http://links.lww.com/PSYMED/A937. Candidates who expressed their interest to participate in the study were first contacted by telephone for an initial screening during which the inclusion criteria were checked and participants were provided with study details. Participants were informed that the study aimed to investigate the effects of intranasal insulin on several blood measures, hunger, and memory. They remained unaware of the specific conditioning hypothesis.

Eligible participants were invited to the laboratory of the Clinical Research Unit of the Leiden University Medical Center for two visits. They were asked to refrain from eating, drinking alcohol and caffeinated drinks, and exercising for a minimum of 12 hours before the study. Patients who received metformin as a treatment were asked not to take it the morning of the study, but they were allowed to take it immediately after the end of the session.

On day 1, upon arrival to the laboratory, participants signed an informed consent form. Their weight and height were measured, and their health status and medication use were assessed. After that, an intravenous catheter was inserted into the median cubital vein by a licensed nurse followed by a baseline blood draw immediately after. Subsequently, participants were asked to smell a fragrant pen for 1 minute by holding the pen approximately 1 cm away from their nose. Immediately thereafter, participants in the conditioned group received 20 units of intranasal insulin spray into one nostril with one puff. Participants in the control group received a placebo spray. Right after administration of the spray, participants were asked to smell the fragrant pen for 1 more minute. Afterward, another sample of blood was drawn. After the blood draw, participants were asked to rate how well they could smell the odor, and their hunger was measured. This procedure of smell-spray-smell administration followed by blood draw and hunger rating was repeated six times every 15 minutes. In between, participants could read a newspaper. After the last spray, participants were given the first part of the memory task. Fifteen minutes after the last spray, the last blood sample was drawn and the catheter was removed. Subsequently, the second part of the memory task was done followed by a mobile food Approach Avoidance Task and a bogus taste test. Day 2 was identical to day 1; however, participants in both conditioned and control groups received a placebo nasal spray. At the end of day 2, participants were fully debriefed about the aims of the study and received a reward of 100 euros.

### Materials

#### Unconditioned Stimulus

The US was 20 units (0.2 ml) of fast-acting insulin (Insulin NovoRapid; Novo Nordisk), administered with the MAD Nasal Intranasal Mucosal Atomization Device (Teleflex) by a trained member of the research team. Six administrations of insulin were done on day 1 in the conditioned group with a break of 15 minutes between the administrations. The spray was administered alternating between the left and then right nostrils. The same dosage of insulin has been successfully used in previous research on insulin conditioning in healthy volunteers (7).

Placebo nasal spray was used in the control group on day 1 and day 2 and in the conditioned group on day 2. The spray was prepared by the Department of Clinical Pharmacy of the Leiden University Medical Center. Because of unavailability of meta-cresol, the preservative that gives a particular smell to the insulin nasal spray, another preservative, chlorobutanol, was used to add a smell to the placebo.

#### Conditioned Stimulus

A smell of rosewood oil was used as a CS. The oil was purchased online from www.aromaolie.nl. This aroma oil has previously been used successfully in a study on classical conditioning of oxytocin (28) by our study group, and, mixed with peppermint oils, in previous research on conditioning of insulin responses (7,9). This smell was rated as pleasant but unfamiliar in previous research (28). Commercially available felt-tip pens were filled with rosewood oil used as a CS. During the smell presentation, participants were asked to hold the pen at approximately 1 cm in front of both nostrils for 1 minute before and 1 minute after the nose spray administration.

### Measurements

Glucose, insulin, and C-peptide levels were measured in blood at baseline, after each spray administration, and 15 minutes after the last spray.

Hunger was measured with a self-rated question, “How hungry do you feel at the moment.” Participants were asked to give an answer on an 11-point numeric rating scale (0, “not hungry at all”; 10, “the worst hunger I have ever experienced”). Hunger was measured at the beginning of each session, 5 minutes after each spray administration, and 20 minutes after the last spray administration.

Approach tendencies toward food were measured at the end of each day with a validated mobile phone approach avoidance task in which participants were presented pictures of food and nonfood objects (29). The task consisted of two blocks: in the congruent block, participants were asked to approach foods by pulling them toward themselves and to avoid objects by pushing them away. In the incongruent block, they were asked to do the opposite—to avoid foods and to approach objects. During each movement, reaction times and response forces were measured. Food approach tendencies are calculated by comparing how fast/strong participants approach foods compared with avoiding them. In total, 80 photographs of food and 40 photographs of objects were presented in a randomized order. During each response, the telephone tracked the gravity- and rotation-corrected acceleration of the movement in the direction perpendicular to the face of the screen (100-Hz sampling rate). Based on this acceleration, two outcome measures were calculated: reaction times (the time between the stimulus presentation and start of response) and force (peak acceleration, in meters per second squared) (29). The pictures for the task were taken from the Food Pics Database (30). The task was presented to the participants on both day 1 and day 2 after the last blood draw.

Food consumption was measured with a taste test adapted from previous studies (31,32). At the end of days 1 and 2, participants were offered several snacks: nuts, cucumbers, blueberries, tomatoes, red pepper, and carrots. They could eat as much as they wanted to. Afterward, the weight of the eaten snacks was measured and the total number of calories eaten was calculated.

Memory was assessed by the Auditory Verbal Learning Test in which 15 words were read to participants 5 times, and participants were asked to repeat all the words they could remember after each reading. Fifteen minutes after the first assessment, participants were asked to name the words they still were able to recall. This is a reliable test for measuring learning and memory (33). Immediate recall scores were calculated by summing the number of all correctly recalled words during the first five assessments. Learning scores were calculated by subtracting the number of the words successfully recalled on the first assessment from the number of the words recalled during the fifth assessment. Percent of forgetting scores was calculated by subtracting the number of words recalled on the delayed recall task from the number of words recalled on the fifth assessment. Version A of the task was given to participants after the last spray administration on day 1 and version B of the task after the last spray administration on day 2.

Perceived group allocation was measured at the end of day 2. Participants were asked to indicate whether they think they received insulin or placebo spray on each of the experimental days.

### Statistical Analysis

The data analyses were performed using SPSS Statistics version 21 (IBM Corporation, Armonk, New York) and RStudio (version 1.1.447; R version 4.0.4). All analyses were performed with a two-tailed significance level of *α* < .05. The data and all analyses codes are available on Open Science Framework (osf.io/nywhq).

A 2 condition (conditioned versus control) × 2 group (healthy versus patient) multivariate analysis of variance was used to compare the groups on the baseline characteristics: age, body mass index, baseline glucose, insulin and C-peptide values, and baseline hunger.

The lmer function of the nlme package in R (R Core Team, 2013) was used for the liner mixed-effects model analyses. Mixed-effects models were applied to the data that included repeated measures (glucose, insulin, C-peptide, hunger, and approach-avoidance task). In all models, the intercept was allowed to vary randomly across participants.

The multilevel structure of the data was defined by measurement time (level 1) nested in participants (level 2). Parameters were estimated using the full-maximum likelihood procedure. In all models, the intercept was allowed to vary randomly across participants. Random slopes did not improve the fit of the models, and therefore, they were removed from the final analysis. The assumption of linearity was checked for each model by plotting the model residuals versus the predictor, and visually inspecting the plots. Homogeneity of variance was checked by the Levene test. Each model was also checked for the normal distribution of its residuals by looking at QQ plots created with Lattice package. In case of violation of any of the assumptions, the data were transformed. The following variables were transformed because of the violation of the homogeneity of variance and nonnormal distribution of the residuals: logarithmic transformation was applied to glucose levels on day 2 and C-peptide levels on day 1 and day 2, the square root transformation was applied to the insulin levels on day 2, and inversion transformation was applied to the reaction time in the approach-avoidance task.

To examine the effects of intranasal insulin administration on blood glucose levels on day 1, a mixed model was performed with day 1 glucose levels as a dependent variable, condition (conditioned versus control), group (healthy versus patient), measurement time (0, 15, 30, 45, 60, 75, or 90 minutes after the first spray administration), baseline glucose levels (measured before the first spray administration), and the interactions between these variables as predictors. To examine the effects of conditioning on blood glucose levels, the same mixed-models analysis was performed but with the measures of day 2. The same analyses were run with insulin, C-peptide, and hunger for each day separately to investigate whether intranasal insulin and conditioning affected these measures. In case an interaction factor was significant, separate models were run for either two groups (healthy and patients) or conditions (conditioned and control), depending on which of the factors was included in this interaction. All mixed models were repeated with sex as a predictor in an exploratory analysis to investigate whether sex affected the relationships between the variables. The effect sizes (Cohen *d*) of all linear mixed-effects models were calculated with EMAtools package. Cohen *d* = 0.2 was interpreted as a small effect size, *d* = 0.5 as a medium effect size, and *d* = 0.8 as a large effect size.

To examine whether intranasal insulin and conditioning affected the approach tendencies toward food, two mixed models were performed. The first model included condition (conditioned versus control), groups (patient versus healthy), day (1 versus 2), stimulus type (food versus object), movement type (pull versus push), and the interaction between these factors as predictors and reaction time as a dependent variable. The second model included the same predictors but movement force as a dependent variable.

A 2 condition (conditioned versus control) × 2 group (healthy versus patient) factorial analysis of variance was used to compare the groups on food consumption during the bogus test: analyses were run separately for day 1 and day 2 with calories eaten as an outcome measure.

A 2 condition (conditioned versus control) × 2 group (healthy versus patient) factorial analysis of variance was used to compare the groups on their memory scores (immediate recall, learning, percentage forgetting). As three separate memory outcomes were used in the analysis, Bonferroni corrections were applied and *α* level was set to .016.

To evaluate success of the blinding, *χ*^2^ test was performed comparing the number of successful guesses with the expected number of successful guesses.

## RESULTS

### Participants

Thirty-two patients with diabetes type 2 (17 men, mean [standard deviation] age = 68.3 [11.86] years) and 32 healthy volunteers (17 men, mean [standard deviation] age = 67.8 [6.12] years) were included in the study. The flowchart with the numbers of screened participants and dropouts is presented in Figure S3, Supplemental Digital Content, http://links.lww.com/PSYMED/A938.

Baseline characteristics are presented in Table 1. There was no difference between conditions (conditioned group versus control) in any baseline characteristic (*F*(10,50) = 0.93, *p* = .52, Wilk Λ = 0.84). Patients had a higher body mass index (*F*(1,63) = 14.86, *p* < .001), higher baseline levels of glucose (*F*(1,63) = 114.32, *p* < .001) and C-peptide (*F*(1,63) = 9.87, *p* < .001) on day 1, higher glucose levels (*F*(1,63) = 91.72, *p* < .001), and C-peptide (*F*(1,63) = 4.95, *p* = .030) on day 2 and higher hunger at baseline on day 1 (*F*(1,63) = 14.61, *p* < .001) than healthy controls.

**TABLE 1:** Baseline Characteristics and Taste Test, Approach-Avoidance Task and Memory Scores With Means, Standard Errors in Parentheses, and Number of Observations in Square Brackets Across Groups and Study Conditions

	Conditioned Group		Control Group	
	Patients	Healthy Controls	Patients	Healthy Controls
Age, y	68.31 (2.37) [16]	67.69 (2.37) [16]	68.20 (2.44) [16]	67.81 (5.5) [16]
Body mass index, kg/m^2^	29.77 (0.84) [16]	25.08 (0.84) [16]	27.77 (0.87) [16]	25.92 (0.84) [16]
Baseline insulin, day 1, mU/L	14.59 (2.17) [16]	9.01 (2.17) [16]	12.69 (2.17) [16]	12.07 (2.17) [16]
Baseline glucose, day 1, mmol/L	8.49 (0.28) [16]	5.35 (0.28) [16]	8.36 (0.28) [16]	5.43 (0.28) [16]
Baseline C-peptide, day 1, mmol/L	1.12 (0.10) [16]	0.74 (0.10) [16]	1.14 (0.10) [16]	0.90 (0.10) [16]
Baseline hunger, day 1	4.5 (0.64) [16]	2.09 (0.64) [16]	5.2 (0.66) [16]	2.69 (0.64) [16]
Baseline insulin, day 2, mU/L	12.8 (2.10) [16]	9.42 (2.10) [16]	10.67 (2.17) [16]	13.11 (2.10) [16]
Baseline glucose, day 2, mmol/L	8.34 (0.30) [16]	5.28 (0.30) [16]	8.18 (0.31) [16]	5.41 (0.30) [16]
Baseline C-peptide, day 2, mmol/L	1.08 (0.10) [16]	0.77 (0.10) [16]	1.07 (0.10) [16]	0.94 (0.10) [16]
Baseline hunger, day 2	3.91 (0.60) [16]	2.59 (0.60) [16]	4.73 (0.62) [16]	4.19 (0.60) [16]
Taste test day 1, kcal	74.99 (15.66) [16]	74.23 (20.56) [16]	142.26 (44.79) [16]	71.59 (17.49) [16]
Taste test day 2, kcal	78.77 (19.72) [16]	89.62 (23.7) [16]	127.45 (37.58) [16]	62.47 (13.95) [16]
Approach to food reaction time, day 1, s*^a^*	0.17 (0.23) [14]	0.14 (0.25) [14]	0.17 (0.31) [15]	0.25 (0.29) [15]
Approach to food reaction time day 2, s*^a^*	0.21 (0.22) [14]	0.25 (0.25) [14]	0.28 (0.24) [15]	0.31 (0.21) [15]
Approach to food force day 1, m/s^2*a*^	0.43 (5.96) [14]	3.93 (6.27) [14]	−1.42 (6.75) [15]	0.91 (7.41) [15]
Approach to food force day 2, m/s^2*a*^	2.29 (6.33) [14]	1.98 (5.95) [14]	0.56 (7.50) [15]	−0.89 (8.48) [15]
Immediate recall day 1	44.60 (2.74) [10]	43.00 (2.50) [12]	40.89 (2.89) [10]	40.42 (2.50) [13]
Immediate recall day 2	45.20 (2.80) [11]	46.25 (2.55) [12]	40.78 (2.95) [10]	41.58 (2.55) [12]
Learning day 1	5.30 (0.56) [10]	5.33 (0.51) [12]	5.67 (0.59) [10]	4.67 (0.51) [13]
Learning day 2	5.90 (0.84) [11]	5.75 (0.76) [12]	4.67 (0.88) [10]	5.33 (0.76) [12]
Percent forgetting day 1	0.21 (0.07) [10]	0.27 (0.06) [12]	0.269 (0.07) [10]	0.164 (0.06) [13]
Percent forgetting day 2	0.24 (0.07) [11]	0.36 (0.06) [12]	0.25 (0.07) [10]	0.22 (0.06) [12]

*^a^* Difference between pull and push conditions.

### Blood Glucose

#### Effects of Insulin Spray (Day 1)

**FIGURE 1:**
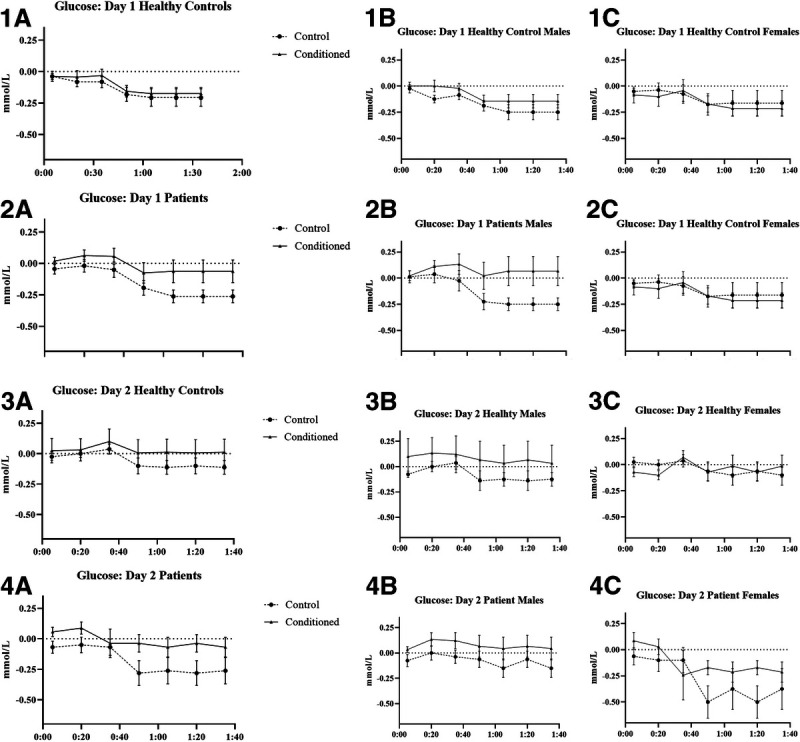
Mean changes of glucose levels from baseline with standard errors.

The effect of time-condition-group interaction (*B* = 0.03, SE = 0.02, *p* = .027, *d* = 0.23) on the blood glucose levels on day 1 was significant. Glucose levels were significantly decreasing with time in healthy participants (*B* = −0.02, SE = 0.01, *p* = .002, *d* = 0.46). In patients, there was a significant time-condition interaction (*B* = 0.03, SE = 0.01, *p* = .011, *d* = 0.37), indicating a significant decrease in glucose levels in patients who received a placebo spray, whereas this decrease was absent in patients who received insulin (Figures 1, 1.A–1.C, 2.A–2.C).

When sex was added to the model as a predictor, a significant time-condition-group-sex interaction was found (*B* = 0.05, SE = 0.02, *p* = .025, *d* = 0.23). There was a significant time-condition interaction in men (*B* = 0.046, SE = 0.02, *p* = .021, *d* = 0.33), indicating that there was a significant decrease in blood glucose levels in men who received placebo, whereas men who received insulin had stable glucose levels (Figures 1.1.A, 1.2.A). The effect of condition (*B* = 0.01, SE = 0.12, *p* = .92, *d* = 0.04) and interactions between condition and other predictors (all *p* values > .54) were insignificant in women.

#### Effects of Conditioning (Day 2)

The effect of time-group interaction (*B* = −0.005, SE = 0.001, *p* = .003, *d* = 0.40) on glucose on day 2 was significant, indicating that there was a decrease in blood glucose levels in both healthy participants (*B* = −0.003, SE = 0.001, *p* = .008, *d* = 0.43) and patients (*B* = −0.01, SE = 0.001, *p* < .001, *d* = 0.92); however, this decrease was more pronounced in patients (Figures 1.3A, 1.4A). Condition (conditioned versus control) did not affect glucose levels on day 2 (*B* = −0.0004, SE = 0.02, *p* = .98, *d* = 0.07).

When sex was added to the model as a predictor, a significant effect of a time-condition-sex interaction (*B* = 0.001, SE = 0.0003, *p* = .024, *d* = 0.23) was found. There was a significant effect of time-condition interaction in men (*B* = −0.02, SE = 0.01, *p* = .024, *d* = 0.32) but not women (*B* = −0.001, SE = 0.03, *p* = .98, *d* = 0.09), indicating that control men had a decrease in blood glucose level, which was absent in conditioned men (Figures 1, 3.A, 3.B, 3.C, 4.A, 4.B, 4.C).

### Insulin

#### Effects of Insulin Spray (Day 1)

There was no effect of condition (insulin versus placebo spray; *B* = −0.07, SE = 0.15, *p* = .67, *d* = 0.11), group (*B* = 0.14, SE = 0.15, *p* = .36, *d* = 0.24), or time (*B* = −0.02, SE = 0.01, *t*(380) = −1.69, *p* = .092, *d* = 0.17) on insulin levels on day 1, neither was the interaction between these factors significant (*B* = 0.05, SE = 0.03, *p* = .084, *d* = 0.18; Figure S4, 1.a, 1.b, Supplemental Digital Content, http://links.lww.com/PSYMED/A939).

There was no significant effect of sex on insulin levels on day 1 (*B* = −0.01, SE = 0.21, *p* = .98, *d* = 0.01); also, the interactions of other variables with sex were not significant (all *p* values > .14).

#### Effects of Conditioning With Insulin (Day 2)

There was no effect of condition (conditioned versus control; *B* = 0.47, SE = 1.03, *p* = .65) or time (*B* = 0.02, SE = 0.09, *p* = .83, *d* = 0.02) on insulin levels on day 2. Patients had significantly higher insulin levels than healthy controls after controlling for baseline levels (*B* = 2.62, SE = 1.03, *p* = .014, *d* = 0.66; Figure S4, 2.a, 2.b, Supplemental Digital Content, http://links.lww.com/PSYMED/A939).

There was no significant effect of sex on insulin levels on day 1 (*B* = −0.81, SE = 1.07, *p* = .45, *d* = 0.20); also, the interactions of other variables with sex were not significant (all *p* values > .41).

### C-Peptide

#### Effects of Insulin Spray (Day 1)

There was a significant effect of the time-condition-group interaction on the C-peptide levels on day 1 (*B* = 0.01, SE = 0.001, *p* = .008, *d* = 0.27). Patients had a significant increase in C-peptide levels during the session (*B* = 0.01, SE = 0.002, *p* = .001, *d* = 0.47). In healthy participants, there was a significant time-condition interaction (*B* = −0.01, SE = 0.003, *p* = .006, *d* = 0.40), demonstrating a decrease in C-peptide levels in healthy participants who received insulin spray, and no change in healthy participants who received placebo (Figure S5, 1.a, 1.b, Supplemental Digital Content, http://links.lww.com/PSYMED/A940).

The time-condition-sex interaction was significant (*B* = 0.04, SE = 0.01, *p* < .001, *d* = 0.51). There was a significant time-condition-group interaction in men (*B* = 0.014, SE = 0.006, *p* = .017, *d* = 0.34), whereas this interaction did not reach significance in women (*B* = 0.007, SE = 0.004, *p* = .056, *d* = 0.29), indicating that the effect found in the whole group was influenced primary by men.

#### Effects of Conditioning With Insulin (Day 2)

There was no effect of condition (*B* = 0.05, SE = 0.05, *p* = .27, *d* = 0.29), group (*B* = 0.05, SE = 0.05, *p* = .26, *d* = 0.30), or time (*B* = 0.001, SE = 0.002, *p* = .83, *d* = 0.02) on the C-peptide levels on day 2 (Figure S5, 2.a, 2.b, Supplemental Digital Content, http://links.lww.com/PSYMED/A940). There was no effect of sex on conditioned C-peptide levels (*B* = −0.002, SE = 0.09, *p* = .98, *d* = 0.006), and the interactions of other variables with sex were not significant (all *p* values > .315).

### Hunger

#### Effects of Insulin Spray (Day 1)

**FIGURE 2:**
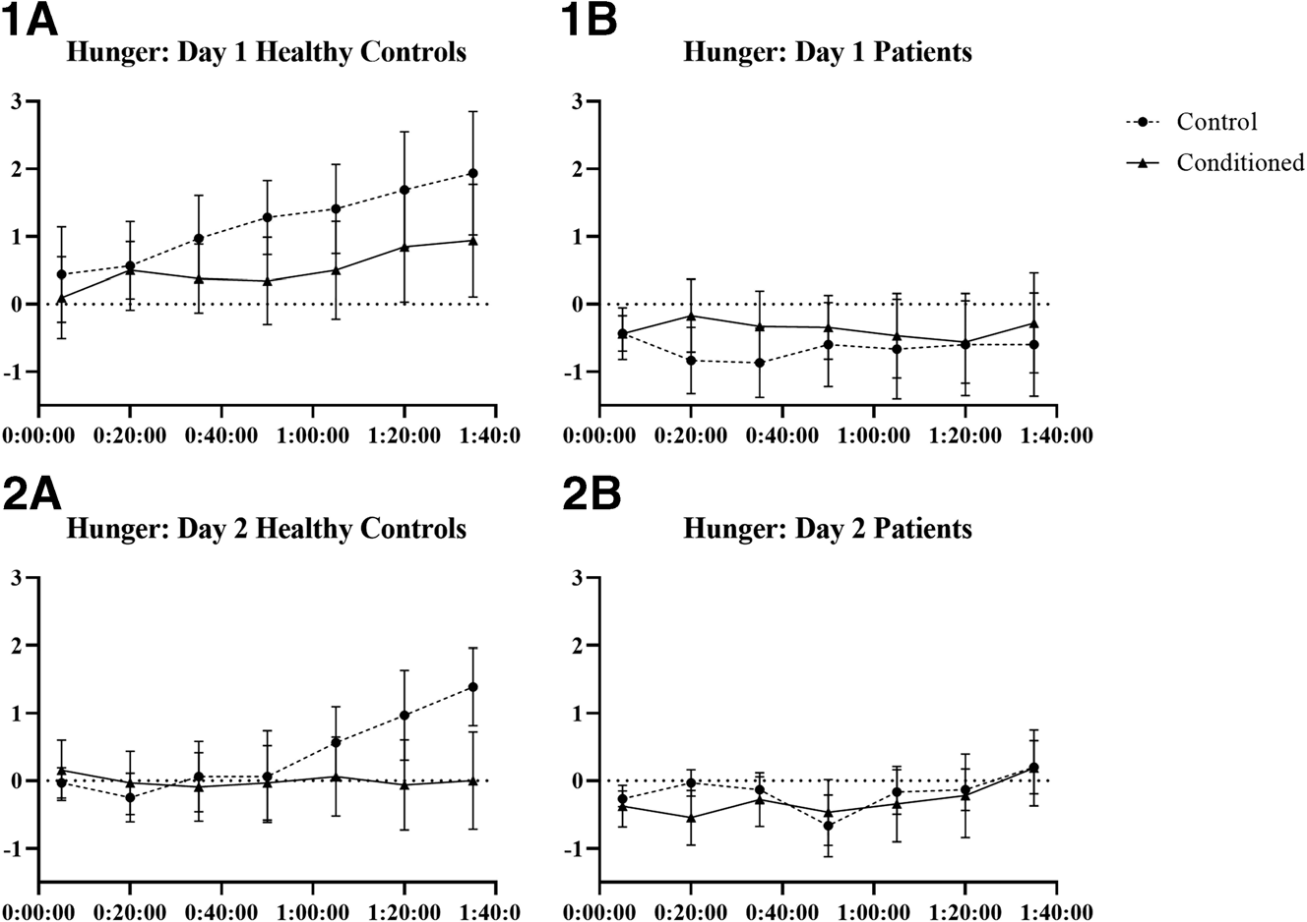
Mean changes of hunger from baseline with standard errors.

There was a significant effect of time (*B* = 0.26, SE = 0.06, *p* < .001, *d* = 0.41) and group-time interaction (*B* = −0.25, SE = 0.09, *p* = .007, *d* = 0.27) on hunger levels on day 1. Hunger increased with time in healthy participants (*B* = 0.26, SE = 0.07, *p* < .001, *d* = 0.53) but stayed stable in patients (*B* = 0.01, SE = 0.06, *p* = .92, *d* = 0.02). There was no effect of condition (insulin versus placebo spray; *B* = −0.46, SE = 0.69, *p* = .50, *d* = 0.17). There was no effect of sex on hunger levels on day 1 (*B* = −0.48, SE = 0.97, *p* = .63, *d* = 0.12), the interactions of other variables with sex were also not significant (all *p* values > .107; Figures 2, 1.A, 1.B).

#### Effects of Conditioning With Insulin (Day 2)

There was a significant effect of time-condition-group interaction (*B* = 0.31, SE = 0.09, *p* < .001, *d* = 0.35) on hunger on day 2. The time-condition interaction was significant in healthy controls (*B* = 0.27, SE = 0.06, *p* < .001, *d* = 0.62) but not in patients (*B* = 0.12, SE = 0.53, *p* = .82, *d* = 0.03), indicating that hunger increased with time in healthy controls in the control group, whereas it stayed stable in the conditioned healthy controls (Figures 2, 2.A, 2.B). When sex was added in the model, the time-condition-group-sex interaction was significant (*B* = −0.42, SE = 0.18, *p* = .011, *d* = 0.27). In men, group-condition-time interaction was significant (*B* = 0.52, SE = 0.12, *p* < .001, *d* = 0.72), indicating an increase in hunger in healthy men from the control group and stable hunger levels in healthy conditioned men and male patients. In women, neither condition (*B* = 0.43, SE = 0.98, *p* = .67, *d* = 0.18) nor any interactions with condition were significant (all *p* values > .23).

### Memory and Food Approach Tendencies

#### Effects of Insulin Spray (Day 1)

There was no effect of intranasal spray administration on the food approach tendencies (reaction time: *B* = −0.01, SE = 0.08, *p* = .93, *d* = 0.16; force: *B* = −2.89, SE = 3.09, *p* = .35, *d* = 0.18), food consumption (*F*(3,62) = 0.75, *p* = .39, *η*^2^ = 0.01), and any of the memory scores (all *p* values > .171). The scores are presented in Table 1, and the results of the analyses of each of the memory scores are presented in Table 2.

**TABLE 2:** The Factorial ANOVAs Comparing Groups and Conditions on Memory Scores

Variable	Factor	*F*	*p*	*η*_p_^2^
Immediate recall day 1	Condition (conditioned versus control)	0.37	.544	0.009
	Group (patients versus healthy controls)	0.02	.885	0.001
	Condition by group	0.003	.960	<0.001
Immediate recall day 2	Condition (conditioned versus control)	1.48	.231	0.035
	Group (patients versus healthy controls)	0.45	.505	0.011
	Condition by group	0.22	.646	0.005
Learning day 1	Condition (conditioned versus control)	0.04	.853	0.001
	Group (patients versus healthy controls)	0.62	.434	0.015
	Condition by group	1.94	.171	0.045
Learning day 2	Condition (conditioned versus control)	0.45	.508	0.011
	Group (patients versus healthy controls)	0.16	.691	0.004
	Condition by group	0.02	.886	0.001
Percent forgetting day 1	Condition (conditioned versus control)	0.19	.666	0.005
	Group (patients versus healthy controls)	0.19	.663	0.005
	Condition by group	1.78	.189	0.043
Percent forgetting day 2	Condition (conditioned versus control)	0.88	.354	0.022
	Group (patients versus healthy controls)	0.43	.515	0.011
	Condition by group	1.15	.290	0.029

ANOVAs = analyses of variance.

#### Effects of Conditioning (Day 2)

There was no effect of conditioning on the food approach tendencies (reaction time: *B* = 0.08, SE = 0.08, *p* = .32, *d* = 0.12; force: *B* = −0.69, SE = 2.95, *p* = .82, *d* < 0.001), food consumption (*F*(3,62) = 1.10, *p* = .23, *η*^2^ = 0.01), and any of the memory scores (all *p* values > .23; Table S1, Supplemental Digital Content, http://links.lww.com/PSYMED/A941).

### Perceived Group Allocation

There was no difference between the conditions in the perceived group allocation (*χ*^2^(1,*N* = 64) = 0.087, *p* = .77), and a majority of participants (90.4%) were unable to correctly guess which spray they received on which day (Table S2, Supplemental Digital Content, http://links.lww.com/PSYMED/A941).

## DISCUSSION

The aim of the current study was to investigate whether it was possible to induce placebo effects in the insulin system through conditioning with intranasal insulin. We studied the effects of conditioning on blood glucose, insulin, and C-peptide levels in patients with diabetes type 2 and healthy controls. In addition, we studied the effects of insulin conditioning on hunger, food consumption, food approach tendencies, and memory. We found that conditioning with intranasal insulin did not affect insulin or C-peptide levels; however, conditioning affected blood glucose levels in men (and not women): men in the conditioned group had higher (i.e., more stable) glucose levels than men in the control group on day 2. This conditioned effects in blood glucose mimicked the action of intranasal insulin, as the same effects were found after the insulin administration on day 1. In addition, we found that conditioning decreased hunger in healthy controls but not in patients with diabetes type 2. We can be certain that the effects found were due to conditioning and not the carryover effects from the previous day, as there were no differences between the baselines of these measures on day 2.

Intranasal insulin administration affected two of the three physiological outcomes of the study: it decreased C-peptide levels in healthy participants and stabilized (prevented from dropping) the glucose levels in patients of both sexes and healthy men. Pharmacologically conditioned effects should normally mimic the effects of the drug, even though in some cases, opposite effects can be found because of negative feedback loops (34). Indeed, we found that the direction of the effects of intranasal insulin administration on blood glucose levels on day 1 corresponded to the direction of the conditioned effects on day 2. As expected, conditioned effects mimicked the effects of the drug; however, the drug affected patients of both sexes and healthy men while conditioning only healthy and patient men. At the same time, the effects of intranasal insulin on C-peptide levels were not successfully conditioned, as no effects of conditioning on C-peptide were found. Regarding the insulin levels, intranasal insulin did not affect endogenous insulin levels; therefore, it is to be expected that conditioning did not affect endogenous insulin levels either.

Importantly, the direction of the effect of intranasal insulin and insulin conditioning on glucose did not correspond to the hypothesized direction found in a previous study (7) that found that both intranasal insulin and conditioning decreased glucose. The main difference between our study and the previous study by Stockhorst and colleagues is the participants’ age: Stockhorst included young healthy men with a mean age of 24 years, whereas our sample consisted of patients and age-matched healthy controls with an average age of 68 years. It is possible that the effects on intranasal insulin may vary with age and health status. Several studies found that various doses of intranasal insulin lead to a mild decrease in blood glucose levels in healthy young adults (35–37), whereas no such effect was found in overweight or obese patients (38) and patients with type 2 diabetes (39). There are multiple changes in energy metabolism occurring with age that are caused by both endocrine changes and changes in lifestyle (40). Therefore, it is quite conceivable that the effects of (conditioning with) intranasal insulin on endocrine and metabolic parameters are different between distinct age groups and people with or without metabolic disease.

It is also hard to say if the conditioned effect we found is beneficial for patients. We did not observe a reduction in blood glucose, which is the primary aim of most diabetes treatments. Conditioning did seem to stabilize blood glucose levels, at least during the test period in men. Instability of plasma glucose levels has been shown to promote microvascular and macrovascular complications such as retinopathy, nephropathy, and heart disease (41,42), and the importance of stabilizing glucose levels is widely discussed in the literature (43,44). Therefore, the effects found in our study may be beneficial for patients; however, it needs to be investigated further.

We have also found that conditioning with intranasal insulin stabilized hunger in healthy participants, partially confirming our study hypothesis. As blood insulin levels rapidly rise after food intake and insulin penetrates the blood-brain barrier, it serves as one of the signals to the central nervous system, and particularly the hypothalamus, to stop feeding and decrease hunger (45). Intranasal insulin has been shown to affect hypothalamic neuronal activity (19). Perhaps conditioning with intranasal insulin triggers neuronal activity in the hypothalamus that dampens appetite. However, this effect was found only in healthy controls and not in patients with diabetes type 2. Patients in our sample did not have any increase in hunger during the sessions, even though they had significantly higher baseline hunger than healthy controls. This finding is in keeping with previous research that found that obese patients and patients with diabetes type 2 might be less responsive to the metabolic effects of intranasal insulin (38,39).

In apparent contrast, no effects of intranasal insulin or conditioning were found on the calories consumed. The total amount of calories eaten during the taste test was very low, possibly because participants knew that the experiment was almost over and they could have a larger meal shortly. For future research, we would propose a more substantial meal, for example, a lunch buffet, to measure food consumption.

No effect of intranasal insulin or insulin conditioning was found on memory. This does not align with several study findings that intranasal insulin administration improves memory in both healthy controls and patients with memory impairments (46,47). However, most of the studies that found memory-improving effects of intranasal insulin investigated the effects of long-term treatment of several weeks (48–50). In our study, we administered 120 units once, which may have been not enough to have an effect on memory. It is worthwhile to investigate whether extending the learning phase of conditioning and administering higher doses of intranasal insulin would lead to conditioned memory improvement.

The sex differences found in our study align with previous research findings of the effects of intranasal insulin. Sex differences were found in previous research on the effects of insulin on declarative and working memory and food intake (51,52); however, not all studies replicated these findings (53). The evidence on sex differentiation in intranasal insulin effects is very mixed to this point and seems to be dependent on the timing of administration and health status of participants. Moreover, it remains unknown whether age-specific sex differences in the responses to intranasal insulin exist and whether they might have played a role in the findings of the present study.

Several limitations of our study must be mentioned. First, it is important to mention that, because of technical issues, we were unable to produce a placebo spray with the same preservative as the insulin spray. Because of this, insulin and placebo sprays had different smells, even though both smells can be described as “medical.” Participants in the conditioned group might have consciously or unconsciously noticed the difference in the way the spray smelled between day 1 and day 2, even though the spray administration was preceded and followed by the administration of a strong smell of aroma oil. However, none of the participants reported noticing the difference, and moreover, when we asked them about the perceived group allocation, a majority of them were not able to correctly tell what spray they received on what day. Despite that, it is important to emphasize that such modification of a part of the CS (that constituted of the rosewood smell, smell of the spray, and the context of spray administration) might possibly have led to a diminished conditioned response. At the same time, we do not expect that this change would completely have blocked the conditioning, as it has been shown that when the CS presented during the evocation phase is slightly different from the CS presented during the acquisition phase, the conditioned effect remains present (54). Second, the findings related to sex differences were done in exploratory analyses, as we had no directional hypothesis regarding sex effects. However, considering sex differences found in previous research, we recruited similar numbers of men and women in each of the experimental groups. A previous experiment documenting a metabolic effect of insulin conditioning (7) studied only men, which matches our findings, showing (albeit opposite) conditioned effects on glucose levels in men only. The impact of sex on the metabolic effects of insulin conditioning needs to be confirmed in a study that is specifically powered to detect sex differences. Third, the men-women ratio in our study is not entirely equal: because of practical issues and the constraints the COVID-19 pandemic posed, we had to deviate slightly from an equal balance. Finally, the results found in our study are not necessarily generalizable to patients with more severe diabetes type 2. We intentionally included only patients with milder disease, who were either with treated behavioral interventions or metformin, and not patients who received insulin injections. Patients with severe insulin resistance or a significant loss of β cells might be less responsive to conditioning manipulations. Finally, it is important to mention that the CS, rosewood oil, might have had certain physiological and psychological effects. Rosewood oil is rich in linalool that has antioxidant and anxiolytic effects (55). However, these effects were found only in studies with administrations of larger doses of linalool (55), and there is not enough evidence that smelling oil for several minutes, as was done in our study, is enough to produce any significant effects. Moreover, as the CS was given in both experimental and control groups, its effects would not be reflected in the between-group comparison.

Our study has several important implications. We demonstrated that conditioning with intranasal insulin reduces hunger in healthy participants. Hunger can be a problem not only for patients with diabetes type 2 but for populations who need to follow a diet for other health reasons. Applying intranasal insulin conditioning can help these groups of people. Importantly, we provided further evidence that glucose responses can be conditioned, not only in healthy controls but also in male patients with diabetes type 2. However, our results indicate that sex and disease-specific effects might play a role in endocrine conditioning, and better understanding of these effects is needed.

## Supplementary Material
