# Supplementary material for: Influencing the Insulin System by Placebo Effects in Patients With Diabetes Type 2 and Healthy Controls: A Randomized Controlled Trial
Source: Psychosom Med. 2023 Jun 23;85(6):551–60. doi: 10.1097/PSY.0000000000001216 (PMC10332649; doi:10.1097/PSY.0000000000001216)
Supplement: Supplementary file 1 [file psymed-85-551-s001.docx]

**Supplemental Digital Content Tables
Table S1.** The factorial ANOVAs comparing groups and conditions on memory scores

| Variable | Factor | F | p | η_p_^2^ |
| --- | --- | --- | --- | --- |
| Immediate recall day 1 | Condition (conditioned versus control) | 0.37 | .544 | .009 |
|  | Group (patients versus healthy controls) | 0.02 | .885 | .001 |
|  | Condition*Group | 0.003 | .960 | <.001 |
| Immediate recall day 2 | Condition (conditioned versus control) | 1.48 | .231 | .035 |
|  | Group (patients versus healthy controls) | 0.45 | .505 | .011 |
|  | Condition*Group | 0.22 | .646 | .005 |
| Learning day 1 | Condition (conditioned versus control) | 0.04 | .853 | .001 |
|  | Group (patients versus healthy controls) | 0.62 | .434 | .015 |
|  | Condition*Group | 1.94 | .171 | .045 |
| Learning day 2 | Condition (conditioned versus control) | 0.45 | .508 | .011 |
|  | Group (patients versus healthy controls) | 0.16 | .691 | .004 |
|  | Condition*Group | 0.02 | .886 | .001 |
| Percent forgetting day 1 | Condition (conditioned versus control) | 0.19 | .666 | .005 |
|  | Group (patients versus healthy controls) | 0.19 | .663 | .005 |
|  | Condition*Group | 1.78 | .189 | .043 |
| Percent forgetting day 2 | Condition (conditioned versus control) | 0.88 | .354 | .022 |
|  | Group (patients versus healthy controls) | 0.43 | .515 | .011 |
|  | Condition*Group | 1.15 | .290 | .029 |

**Table S2.** Number of correct and incorrect guesses regarding the group allocation per group.

|  | Condition | |
| --- | --- | --- |
|  | Conditioned | Control |
| Correct guess | 8 | 7 |
| Incorrect guess | 24 | 25 |
