# Supplementary material for: Influencing the Insulin System by Placebo Effects in Patients With Diabetes Type 2 and Healthy Controls: A Randomized Controlled Trial
Source: Psychosom Med. 2023 Jun 23;85(6):551–60. doi: 10.1097/PSY.0000000000001216 (PMC10332649; doi:10.1097/PSY.0000000000001216)
Supplement: Supplementary file 2 [file psymed-85-551-s002.docx]

**Influencing the insulin system by placebo effects in patients with diabetes type-2 and healthy controls: A randomized controlled trial**

Aleksandrina Skvortsova^1,2*^, PhD; Dieuwke S. Veldhuijzen^2^, PhD; Lotte F. van Dillen^3^, PhD; Hilmar Zech^2,4^, MSc; Suzanne M.J.C. Derksen^2^, MSc; Ruben H. Sars^2^, MSc; Onno C. Meijer^5^, PhD; Hanno Pijl^5^, PhD; Andrea WM Evers^2,6,7^, PhD.

1. Department of Psychology, McGill University, Canada; 2. Health, Medical and Neuropsychology unit, Faculty of Social and Behavioural Sciences, Leiden University, the Netherlands; 3. Social, Economic and Organizational Psychology unit, Faculty of Social and Behavioural Sciences, Leiden University, the Netherlands; 4. Neuroimaging center, Dresden University of Technology, Germany; 5. Department of Medicine, Division of Endocrinology, Leiden University Medical Centre, Leiden, the Netherlands; 6. Department of Psychiatry, Leiden University Medical Center, Leiden, the Netherlands; 7. Medical Delta, Leiden University, Technical University Delft and Erasmus University, the Netherlands.

*Corresponding author

Email: [a.skvortsova@fsw.leidenuniv.nl](mailto:a.skvortsova@fsw.leidenuniv.nl)

**Conflicts of Interest and Source of Funding.** The study was funded by the Diabetes II Breakthrough grant by Dutch Diabetes Fonds and ZonMW (project number 459001009) awarded to AWME. The work of HZ was supported by a grant from the German Research Foundation (project number 402170461). The authors have no conflicts of interest.

**Previous Posting:** This manuscript was posted as a preprint on OSF on May 16, 2022, <https://doi.org/10.31219/osf.io/3ru6q>

Number of Figures= 2; Number of Tables= 1; Number of SDC files = 6 (.docx, .png, .jpg)
 **Article Editor:** Julian F. Thayer

**Abstract**

**Objective.** The objective of this study was to investigate whether placebo effect induced by pharmacological conditioning with intranasal insulin can affect glucose, insulin, c-peptide, hunger and memory in patients with diabetes type-2 and healthy controls.

**Methods.** Placebo effect was induced by pharmacological conditioning. Thirty-two older patients (mean age= 68.3) with diabetes type-2 and age and sex matched thirty-two healthy older adults (mean age=67.8) were randomly assigned to a conditioned or a control group. On day 1, conditioned group received 6 administrations of intranasal insulin with a CS (smell of rosewood oil) while the control group received a placebo with the CS. On day 2, both groups received a placebo spray with the CS. Glucose, insulin and c-peptide were repeatedly measured in blood. Hunger and memory were assessed with validated measures.

**Results.** Intranasal insulin stabilized dropping glucose levels in patients (B=0.03, SE=0.02, p=0.027) and healthy men (B=0.046, SE=0.02, p=0.021), and decreased C-peptide levels in healthy controls (B=0.01, SE=0.001, p=0.008). Conditioning also prevented the drop of glucose levels but only in men (both healthy and patients) (B=0.001, SE=0.0003, p=0.024). Conditioning significantly decreased hunger in healthy participants (B=0.31, SE=0.09, p<0.001). No effects were found on other measures.

**Conclusions.** Placebo effect induced by conditioning with intranasal insulin modify blood glucose levels and decrease hunger in older adults but its effects depend on health status and sex. Insulin conditioning might be beneficial for groups suffering from intensive hunger but seems not be particularly suitable for blood glucose reduction.

**Trial registration.** Netherlands Trial Register, NL7783 (<https://www.trialregister.nl/trial/7783>).

**Key words:** pharmacological conditioning, placebo effect, intranasal insulin, type-2 diabetes, glucose

Abbreviations: CS- conditioned stimulus; US- unconditioned stimulus

1. **Introduction**

Placebo effects are positive treatments outcomes that cannot be attributed to the pharmacological mechanisms of the treatment but are caused by the psychosocial context (1). Placebo effects can be induced by positive patient-doctor communication, observational learning or associative leaning through classical conditioning procedures. Accumulating evidence suggests that it is possible to modulate endocrine functions using classical conditioning (2, 3): coupling of an active medication (unconditioned stimulus, US) with an initially neutral stimulus (conditioned stimulus, CS). In case of endocrine conditioning, hormonal-stimulating or inhibiting medication (US) gets associated with the CS, and later the mere presentation of the CS alone leads to changes in hormone levels or triggers effects associated with this hormone.

Several possible clinical applications of placebo effects induced by conditioning were proposed (4, 5). For example, dosages of standard treatments can be reduced using placebo-controlled dose reduction protocols, in which an active drug gets pharmacologically conditioned and then a part of it is replaced by a placebo while maintaining the efficacy of treatment (4). Placebo effects can also boost the efficiency of treatments when conditioning procedures are added to the standard treatment protocols (6).

The most convincing evidence for endocrine conditioning comes from studies on conditioning of insulin and glucose responses in animals and healthy humans (7-11). Insulin and glucose responses seem to be particularly malleable by the mechanisms of conditioning (12), probably due to their acute homeostatic functions aimed at maintaining glucose metabolism. Cephalic phase release of insulin, for example, is a transient pulse of insulin, that has been observed in both animals and humans in response to food cues, such as the smell of food, or the time of the day when food is regularly taken (12). This conditioned response seems to help prepare the organism for the upcoming homeostatic changes related to the food consumption and prevent hyperglycemia caused by consumption of large amounts of food (13). Not only naturally occurring associations, such as associations between the smell of food and food intake, can trigger conditioned insulin responses. Experimental studies demonstrated that coupling of food with any neutral stimuli, such as a sound or a light, can trigger conditioned insulin release (14-16). Moreover, insulin and glucose responses can be conditioned using US other than food. Using insulin injections as a US, it was found possible to classically condition glucose decrease in healthy young volunteers (9, 10). Another study successfully conditioned insulin release and glucose decrease in healthy volunteers using intranasal insulin administration as a US (7).

Up to date, most of the animal research on insulin conditioning has been done in male mice or rats (for the review see 2) and the few available human studies were performed in young male volunteers. Therefore, it remains unknown whether sex or age might play any role in the conditionability of insulin effects. Importantly, there are no reports of the possibility to condition insulin responses in metabolic disorders. Particularly, patients with diabetes type-2 might benefit from conditioning with intranasal insulin as an unconditioned stimulus because intranasal insulin has been shown to have a number of benefits for patients with diabetes type-2. Conditioning with insulin might trigger conditioned insulin release and glucose decrease (7) without causing common side effects of intravenous insulin injections such as hypoglycaemia and hypertension (17). Moreover, since intranasal insulin normalizes hypothalamic neuronal activity in response to glucose ingestion, it could be especially favorable for type-2 diabetes patients who demonstrate distorted brain responses to glucose (18, 19). Additionally, evidence suggests that intranasal insulin decreases food intake and hunger (20, 21), and improves memory both in healthy volunteers and patients with diabetes type-2 (22, 23). Taken together, classical conditioning with intranasal insulin has a wide range of potential positive effects for patients with diabetes type-2.

The aim of the present study was to investigate the effects of conditioning with intranasal insulin on blood glucose, insulin, C-peptide, hunger and memory in a group of diabetes type-2 patients and age and sex-matched healthy controls. Additionally, we aimed to explore differences between healthy individuals and patients with diabetes type-2 as their responses to insulin and conditioning might differ due to insulin resistance (24) or different baseline levels of glucose (25) or metabolic hormones (26, 27). Finally, we explored possible sex differences in the effects of conditioning with intranasal insulin.

1. **Methods**
   1. **Participants**

Patients diagnosed with diabetes type-2 and healthy controls were included in the study. Healthy controls were matched for age (the mean age of the groups was matched +/-1 year) and sex to the patients group. Inclusion criteria for the patients were: 1) being older than 18 years old; 2) current diagnosis of diabetes type-2; 3) taking metformin and/or participating in a lifestyle intervention (e.g., diet) to control their diabetes. Exclusion criteria for both healthy subjects and patients were: 1) use of insulin or insulin stimulating medications; 2) use of medication that influences glucose metabolism (for example, corticosteroid medication, chemotherapy, beta-blockers); 3) diagnosis of a chronic non-communicable disease (degenerative diseases, malignant neoplasms such as cancer, diabetes type-1, auto-immune diseases); 4) diagnosis of an acute infectious disease (such as meningitis, hepatitis B, bacterial pneumonia); 5) current diagnosis of a mental disorder; 6) chronic and/or acute rhinitis, 7) anatomic deviations of the nose; 8) substance abuse (e.g., drugs or alcohol); 9) pregnancy.

The sample size calculation was based on the results of the study with a comparable design in healthy participants which reported an effect size of d=0.77 (7). A power analysis using this effect size yielded that 16 participants per condition and per group are needed with a power of 0.8 and a two-sided alpha of 0.05 as determined by G*Power software.

- 1. **Study design**

The study had a double-blind randomized placebo-controlled design. Thirty-two patients with diabetes type-2 and thirty-two healthy controls were randomized to one of two groups in a double-blind manner: 1) conditioned group; 2) control group. Men and women were equally distributed between the groups. This study was an adaptation of the study design used by Stockhorst and colleagues (7) for conditioning insulin responses in healthy participants. The study conditions are presented in Figure S1, Supplemental Digital Content, http://links.lww.com/PSYMED/A936.

The study was approved by the Medical Ethical Committee of Leiden, Den Haag, Delft under protocol number P18.222.

Randomization was performed by the Department of Clinical Pharmacy of the Leiden University Medical Center. A block randomization was used with a size of eight participants per block. Equal numbers of men and women were randomized to each condition. The pharmacy was responsible for assigning participants to the conditions. The experimenter was blinded regarding the conditions and the list coupling participants numbers with conditions remained at the pharmacy until the last participant was tested.

- 1. **Procedure**

The data collection was done from May 2019 until March 2021. The study procedures are presented in Figure S2, Supplemental Digital Content, http://links.lww.com/PSYMED/A937. Candidates who expressed their interest to participate in the study were first contacted by phone for an initial screening during which inclusion criteria were checked and participants were provided with study details. Participants were informed that the study aimed to investigate the effects of intranasal insulin on several blood measures, hunger and memory. They remained unaware of the specific conditioning hypothesis.

Eligible participants were invited to the laboratory of the Clinical Research Unit of the Leiden University Medical Center for two visits. They were asked to refrain from eating, drinking alcohol and caffeinated drinks and exercising for a minimum of 12 hours before the study. Patients, who received metformin as a treatment, were asked not to take it the morning of the study but they were allowed to take it immediately after the end of the session.

On day 1, upon arrival to the lab, participants signed an informed consent form. Their weight and height were measured, and their health status and medication use were assessed. After that, an intravenous catheter was inserted into the median cubital vein by a licensed nurse followed by a baseline blood draw immediately after. Subsequently, participants were asked to smell a fragrant pen for one minute by holding the pen approximately 1 cm away from their nose. Immediately thereafter, participants in the conditioned group received 20 units of intranasal insulin spray into one nostril with one puff. Participants in the control group received a placebo spray. Right after administration of the spray, participants were asked to smell the fragrant pen for one more minute. Afterwards, another sample of blood was drawn. After the blood draw, participants were asked to rate how well the could smell the odor, and their hunger was measured. This procedure of smell-spray-smell administration followed by blood draw and hunger rating was repeated 6 times every 15 minutes. In between, participants could read a newspaper. After the last spray, participants were given the first part of the memory task. 15 minutes after the last spray, the last blood sample was drawn and the catheter was removed. Subsequently, the second part of the memory task was done followed by a mobile food Approach Avoidance Task and a bogus taste test. Day 2 was identical to day 1, however, participants in both conditioned and control groups, received a placebo nasal spray. At the end of the day 2, participants were fully debriefed about the aims of the study and received a reward of 100 euros.

- 1. **Materials**
     1. ***Unconditioned stimulus***

The unconditioned stimulus was 20 units (0.2 ml) of fast-acting insulin (Insulin NovoRapid; Novo Nordisk), administered with the MAD Nasal Intranasal Mucosal Atomization Device (Teleflex) by a trained member of the research team. Six administrations of insulin were done on day 1 in the conditioned group with a break of 15 minutes between the administrations. The spray was administered alternating between the left and then right nostrils. The same dosage of insulin has been successfully used in previous research on insulin conditioning in healthy volunteers (7).

Placebo nasal spray was used in the control group on day 1 and day 2 and on day 2 in in the conditioned group. The spray was prepared by the Department of Clinical Pharmacy of the Leiden University Medical Center. Because of unavailability of meta-cresol, the preservative that gives a particular smell to the insulin nasal spray, another preservative, chlorobutanol, was used to add a smell to the placebo.

- - 1. ***Conditioned stimulus***

A smell of rosewood oil was used as a CS. The oil was purchased online from www.aromaolie.nl. This aroma oil has previously been used successfully in a study on classical conditioning of oxytocin (28) by our study group, and, mixed with peppermint oils, in previous research on conditioning of insulin responses (7, 9). This smell was rated as pleasant but unfamiliar in previous research (28). Commercially available felt-tip pens were filled with rosewood oil used as a CS. During the smell presentation, participants were asked to hold the pen at approximately 1 cm in front of both nostrils for one minute before and one minute after the nose spray administration.

- 1. **Measurements**

*Glucose, insulin, and c-peptide* levels were measured in blood at baseline, after each spray administration and 15 min after the last spray.

*Hunger* was measured with a self-rated question “How hungry do you feel at the moment”. Participants were asked to give an answer on a 11-point numeric rating scale (0- “not hungry at all”; 10- “the worst hunger I have ever experienced”). Hunger was measured at the beginning of each session, 5 minutes after each spray administration and 20 minutes after the last spray administration.

*Approach tendencies towards food* were measured at the end of each day with a validated mobile phone approach avoidance task in which participants were presented pictures of food and non-food objects (29). The task consisted of two blocks: in the congruent block, participants were asked to approach foods by pulling them towards themselves and to avoid objects by pushing them away. In the incongruent block, they were asked to do the opposite—to avoid foods and to approach objects. During each movement reaction times and response forces were measured. Food approach tendencies are calculated by comparing how fast/strong participants approach foods compared to avoiding them. In total, 80 photos of food and 40 photos of objects were presented in a randomized order. During each response, the phone tracked the gravity- and rotation-corrected acceleration of the movement in the direction perpendicular to the face of the screen (100Hz sampling rate). Based on this acceleration, two outcome measures were calculated: reaction times (the time between the stimulus presentation and start of response) and force (peak acceleration, meters/seconds^2) (29). The pictures for the task were taken from the Food Pics Database (30). The task was presented to the participants on both day 1 and day 2 after the last blood draw.

*Food consumption* was measured with a taste test adapted from previous studies (31, 32). At the end of day 1 and 2, participants were offered several snacks: nuts, cucumbers, blueberries, tomatoes, red pepper and carrots. They could eat as much as they wanted to. Afterwards, the weight of the eaten snacks was measured and the total number of calories eaten was calculated.

*Memory* was assessed by the Auditory Verbal Learning Test in which 15 words were read to participants 5 times and participants were asked to repeat all the words they could remember after each reading. Fifteen minutes after the first assessment participants were asked to name the words they still were able to recall. This is a reliable test for measuring learning and memory (33). Immediate recall scores were calculated by summing the number of all correctly recalled words during the first 5 assessments. Learning scores were calculated by subtracting the number of the words successfully recalled on the first assessment from the number of the words recalled during the fifth assessment. Percent of forgetting scores were calculated by subtracting the number of words recalled on the delayed recall task from the number of words recalled on the fifth assessment. Version A of the task was given to participants after the last spray administration of day 1 and version B of the task after the last spray administration of day 2.

*Perceived group allocation* was measured at the end of the day 2. Participants were asked to indicate whether they think they received insulin or placebo spray on each of the experimental days.

- 1. **Statistical analysis**

The data analyses were performed using SPSS Statistics version 21 (IBM Corporation, Armonk, NY) and RStudio (version 1.1.447; R version 4.0.4). All analyses were performed with a 2-tailed significance level of α <.05. The data and all analyses codes are available on Open Science Framework (osf.io/nywhq).

A 2 condition (conditioned vs control) x 2 group (healthy vs patient) multivariate analysis of variance was used to compare the groups on the baseline characteristics: age, body mass index, baseline glucose, insulin and c-peptide values and baseline hunger.

The lmer function of the nlme package in R (R Core Team, 2013) was used for the liner mixed effects models analyses. Mixed effects models were applied to the data that included repeated measures (glucose, insulin, C-peptide, hunger and approach-avoidance task). In all models, the intercept was allowed to vary randomly across participants.

The multilevel structure of the data was defined by measurement time (level 1) nested in participants (level 2). Parameters were estimated using the full maximum likelihood procedure. In all models, the intercept was allowed to vary randomly across participants. Random slopes did not improve the fit of the models and, therefore, they were removed from the final analysis. The assumption of linearity was checked for each model by plotting the model residuals versus the predictor, and visually inspecting the plots. Homogeneity of variance was checked by Levene’s test. Each model was also checked for the normal distribution of its residuals by looking at QQ plots created with Lattice package. In case of violation of any of the assumptions, the data were transformed. The following variables were transformed due to the violation of the homogeneity of variance and non-normal distribution of the residuals: logarithmic transformation was applied to glucose levels of the day 2, C-peptide levels of the day 1 and day 2, the square root transformation was applied to the insulin levels of day 2, inversion transformation was applied to the reaction time in the approach-avoidance task.

To examine the effects of intranasal insulin administration on blood glucose levels on day 1, a mixed model was performed with day 1 glucose levels as a dependent variable, condition (conditioned vs control), group (healthy vs patient), measurement time (0, 15, 30, 45, 60, 75 or 90 minutes after the first spray administration), baseline glucose levels (measured before the first spray administration) and the interactions between these variables as predictors. To examine the effects of conditioning on blood glucose levels, the same mixed model analysis was performed but with the measures of the day 2. The same analyses were run with insulin, C-peptide and hunger for each day separately to investigate whether intranasal insulin and conditioning affected these measures. In case an interaction factor was significant, separate models were run for either two groups (healthy and patients) or conditions (conditioned and control) depending on which of the factors was included in this interaction. All mixed models were repeated with sex as a predictor in an exploratory analysis to investigate whether sex affected the relationships between the variables. The effect sizes (Cohen’s d) of all linear mixed effects models were calculated with EMAtools package. Cohen’s d=0.2 was interpreted as a small effect size, d=0.5 as a medium effect size, and d=0.8 as a large effect size (34).

To examine whether intranasal insulin and conditioning affected the approach tendencies towards food, two mixed models were performed. The first model included condition (conditioned vs control), groups (patient vs healthy), day (1 vs 2), stimulus type (food versus object), movement type (pull versus push) and the interaction between these factors as predictors and reaction time as a dependent variable. The second model included the same predictors but movement force as a dependent variable.

A 2 condition (conditioned vs control) x 2 group (healthy vs patient) factorial analysis of variance (ANOVA) was used to compare the groups on food consumption during the bogus test: analyses were run separately for day 1 and day 2 with calories eaten as an outcome measure.

A 2 condition (conditioned vs control) x 2 group (healthy vs patient) factorial ANOVA was used to compare the groups on their memory scores (immediate recall, learning, percentage forgetting). As three separate memory outcomes were used in the analysis, Bonferroni corrections were applied and alpha level was set to 0.016.

To evaluate success of the blinding, χ^2^ test was performed comparing the number of successful guesses to the expected number of successful guesses.

1. **Results**
   1. **Participants**

Thirty-two patients with diabetes type-2 (17 men, mean age=68.3, SD=11.86) and thirty-two healthy volunteers (17 men, mean age=67.8, SD=6.12) were included in the study. The flowchart with the numbers of screened participants and dropouts is presented in Figure S3, Supplemental Digital Content, http://links.lww.com/PSYMED/A938.

Baseline characteristics are presented in Table 1. There was no difference between conditions (conditioned group versus control) in any baseline characteristic (F(10,50)=0.93, p=0.52, Wilk's Λ=0.84). Patients had a higher BMI (F(1,63)=14.86, p< .001), higher baseline levels of glucose (F(1,63)=114.32, p< .001) and c-peptide (F(1,63)=9.87, p< .001) on day 1, higher glucose levels (F(1,63)=91.72, p< .001) and c-peptide (F(1,63)=4.95, p=.030) on day 2 and higher hunger at baseline on day 1 (F(1,63)=14.61, p< .001) than healthy controls.

- 1. **Blood glucose**

*Effects of insulin spray (Day 1).* The effect of time-condition-group interaction (B=0.03, SE=0.02, p=.027, d=0.23) on the blood glucose levels on Day 1 was significant. Glucose levels were significantly decreasing with time in healthy participants (B=-0.02, SE=0.01, p=.002, d=0.46). In patients, there was a significant time-condition interaction (B=0.03, SE=0.01, p=.011, d=0.37), indicating a significant decrease in glucose levels in patients who received a placebo spray while this decrease was absent in patients who received insulin (Figure 1, 1.a, 1.b, 1.c, 2.a, 2.b, 2.c).

When sex was added to the model as a predictor, a significant time-condition-group-sex interaction was found (B=0.05, SE=0.02, p=.025, d=0.23). There was a significant time-condition interaction in men (B=0.046, SE=0.02, p=.021, d=0.33), indicating that there was a significant decrease in blood glucose levels in men who received placebo, while males who received insulin had stable glucose levels (Figure 1.1, 2.2). The effect of condition (B=0.01, SE=0.12, p=.92, d=0.04) and interactions between condition and other predictors (all p values > 0.54) were insignificant in women.

*Effects of conditioning (Day 2).* The effect of time-group interaction (B=-0.005, SE=0.001, p=.003, d=0.40) on glucose on Day 2 was significant, indicating that there was a decrease in blood glucose levels in both healthy participants (B=-0.003, SE=0.001, p=.008, d=0.43) and patients (B=-0.01, SE=0.001, p< 0.001, d=0.92), however, this decrease was more pronounced in patients (Figure 1.3, 2.4). Condition (conditioned vs control) did not affect glucose levels on day 2 (B=-0.0004, SE=0.02, p=.98, d=0.07).

When sex was added to the model as a predictor, a significant effect of a time-condition-sex interaction (B=0.001, SE=0.0003, p=.024, d=0.23) was found. There was a significant effect of time-condition interaction in men (B=-0.02, SE=0.01, p=.024, d=0.32) but not women (B=-0.001, SE=0.03, p=.98, d=0.09), indicating that control men had a decrease in blood glucose level, that was absent in conditioned men (Figure 1, 3.a, 3.b, 3.c, 4.a, 4.b, 4.c).

- 1. **Insulin**

*Effects of insulin spray (Day 1).* There was no effect of condition (insulin versus placebo spray) (B=-0.07, SE=0.15, p=.67, d=0.11), group (B=0.14, SE=0.15, p=.36, d=0.24) or time (B=-0.02, SE=0.01, t(380)=-1.69, p=.092, d=0.17) on insulin levels on day 1, neither was the interaction between these factors significant (B=0.05, SE=0.03, p=.084, d=0.18) (Figure S4, 1.a, 1.b, Supplemental Digital Content, http://links.lww.com/PSYMED/A939).

There was no significant effect of sex on insulin levels on day 1 (B=-0.01, SE=0.21, p=.98, d=0.01), also the interactions of other variables with sex were not significant (all p values >.14).

*Effects of conditioning with insulin (Day 2)*. There was no effect of condition (conditioned versus control) (B=0.47, SE=1.03, p=.65) or time (B=0.02, SE=0.09, p=.83, d=0.02) on insulin levels on day 2. Patients had significantly higher insulin levels than healthy controls after controlling for baseline levels (B=2.62, SE=1.03, p=.014, d=0.66) (Figure S4, 2.a, 2.b).

There was no significant effect of sex on insulin levels on day 1 (B=-0.81, SE=1.07, p=.45, d=0.20), also the interactions of other variables with sex were not significant (all p values >.41).

- 1. **C-peptide**

*Effects of insulin spray (Day 1).* There was a significant effect of the time-condition-group interaction on the C-Peptide levels on day 1 (B=0.01, SE=0.001, p=.008, d=0.27). Patients had a significant increase in C-peptide levels during the session (B=0.01, SE=0.002, p=.001, d=0.47). In healthy participants, there was a significant time-condition interaction (B=-0.01, SE=0.003, p=.006, d=0.40), demonstrating a decrease in C-peptide levels in heathy participants who received insulin spray, and no change in healthy participants who received placebo (Figure S5, 1.a, 1.b, Supplemental Digital Content, http://links.lww.com/PSYMED/A940).

The time-condition-sex interaction was significant (B=0.04, SE=0.01, p < 0.001, d=0.51). There was a significant time-condition-group interaction in men (B=0.014, SE=0.006, p=.017, d=0.34) while this interaction did not reach significance in women (B=0.007, SE=0.004, p= .056, d=0.29), indicating that the effect found in the whole group was influenced primary by men.

*Effects of conditioning with insulin (Day 2)*. There was no effect of condition (B=0.05, SE=0.05, p=.27, d=0.29), group (B=0.05, SE=0.05, p=.26, d=0.30) or time (B=0.001, SE=0.002, p=.83, d=0.02) on the C-peptide levels on day 2 (Figure S5, 2.a, 2.b). There was no effect of sex on conditioned C-peptide levels (B=-0.002, SE=0.09, p=.98, d=0.006), the interactions of other variables with sex were not significant (all p values > .315).

- 1. **Hunger**

*Effects of insulin spray (Day 1).* There was a significant effect of time (B=0.26, SE=0.06, p< .001, d=0.41) and group-time interaction (B=-0.25, SE=0.09, p=.007, d=0.27) on hunger levels on day 1. Hunger increased with time in healthy participants (B=0.26, SE=0.07, p < .001, d=0.53) but stayed stable in patients (B=0.01, SE=0.06, p=.92, d=0.02). There was no effect of condition (insulin versus placebo spray) (B=-0.46, SE=0.69, p=.50, d=0.17). There was no effect of sex on hunger levels on day 1 (B=-0.48, SE=0.97, p=.63, d=0.12), the interactions of other variables with sex were also not significant (all p values > 0.107) (Figure 2, 1.a, 1.b).

*Effects of conditioning with insulin (Day 2)*. There was a significant effect of time-condition- group interaction (B=0.31, SE=0.09, p < .001, d=0.35) on hunger on day 2. The time-condition interaction was significant in healthy controls (B=0.27, SE=0.06, p < .001, d=0.62) but not in patients (B=0.12, SE=0.53, p=.82, d=0.03) indicating that hunger increased with time in healthy controls in the control group while it stayed stable in the conditioned healthy controls (Figure 2, 2.a, 2.b). When sex was added in the model, the time-condition-group-sex interaction was significant (B=-0.42, SE=0.18, p=.011, d=0.27). In men, group-condition-time interaction was significant (B=0.52, SE=0.12, p<.001, d=0.72), indicating increase in hunger in healthy men from the control group and stable hunger levels in healthy conditioned men and male patients. In women neither condition (B=0.43, SE=0.98, p=.67, d=0.18), nor any interactions with condition were significant (all p values >0.23).

- 1. **Memory & food approach tendencies**

*Effects of insulin spray (Day 1).* There was no effect of intranasal spray administration on the food approach tendencies (reaction time: B=-0.01, SE=0.08, p=0.93, d=0.16; force: B=-2.89, SE=3.09, p=.35, d=0.18), food consumption (F(3,62)=.75, p=.39, η^2^= 0.01), and any of the memory scores (all p values > .171). ). The scores are presented in Table 1 and the results of the analyses of each of the memory scores are presented in Table 2.

*Effects of conditioning (Day 2).* There was no effect of conditioning on the food approach tendencies (reaction time: B=0.08, SE=0.08, p=.32, d=0.12; force: B=-0.69, SE=2.95, p=.82, d< 0.001), food consumption (F(3,62)=1.10, p=.23, η^2^= 0.01), and any of the memory scores (all p values > .23) (Table S1, Supplemental Digital Content, http://links.lww.com/PSYMED/A941).

- 1. **Perceived group allocation**

There was no difference between the conditions in the perceived group allocation (χ^2^(1, N = 64) = .087, p = .77) and the majority of participants (90.4%) were unable to correctly guess which spray they received on which day (Table S2, Supplemental Digital Content, http://links.lww.com/PSYMED/A941).

1. **Discussion**

The aim of the current study was to investigate whether it was possible to induce placebo effects in the insulin system through conditioning with intranasal insulin. We studied the effects of conditioning on blood glucose, insulin, and c-peptide levels in patients with diabetes type-2 and healthy controls. Additionally, we studied the effects of insulin conditioning on hunger, food consumption, food approach tendencies and memory. We found that conditioning with intranasal insulin did not affect insulin or C-peptide levels, however, conditioning affected blood glucose levels in men (and not women): men in the conditioned group had higher (i.e., more stable) glucose levels than men in the control group on day 2. This conditioned effects in blood glucose mimicked the action of intranasal insulin, as the same effects were found after the insulin administration on day 1. Additionally, we found that conditioning decreased hunger in healthy controls, but not in patients with diabetes type-2. We can be certain that the effects found were due to conditioning and not the carry-over effects from the previous day, as there were no differences between the baselines of these measures on day 2.

Intranasal insulin administration affected two of the three physiological outcomes of the study: it decreased C-peptide levels in healthy participants and stabilized (prevented from dropping) the glucose levels in patients of both sexes and healthy men. Pharmacologically conditioned effects should normally mimic the effects of the drug, even though in some cases opposite effects can be found due to negative feedback loops (35). Indeed, we found that the direction of the effects of intranasal insulin administration on blood glucose levels on day 1 corresponded to the direction of the conditioned effects on day 2. As expected, conditioned effects mimicked the effects of the drug, however, the drug affected patients of both sexes and healthy men, while conditioning- only healthy and patient men. At the same time, the effects of intranasal insulin on C-peptide levels were not successfully conditioned as no effects of conditioning on C-peptide was found. Regarding the insulin levels, intranasal insulin did not affect endogenous insulin levels, therefore, it is to be expected that conditioning did not affect endogenous insulin levels either.

Importantly, the direction of the effect of intranasal insulin and insulin conditioning on glucose did not correspond to the hypothesized direction found in a previous study (7) that found that both intranasal insulin and conditioning decreased glucose. The main difference between our study and the previous study by Stockhorst and colleagues is the participants’ age: Stockhorst included young healthy men with a mean age of 24 years while our sample consisted of patients and age-matched healthy controls with an average age of 68 years old. It is possible that the effects on intranasal insulin may vary with age and health status. Several studies found that various doses of intranasal insulin lead to a mild decrease of blood glucose levels in healthy young adults (36, 37, 38) while no such effect was found in overweight or obese patients (39) and patients with type-2 diabetes (40). There are multiple changes in energy metabolism occurring with age that are caused by both endocrine changes and changes in lifestyle (41). Therefore, it is quite conceivable that the effects of (conditioning with) intranasal insulin on endocrine and metabolic parameters are different between distinct age groups and people with or without metabolic disease.

It is also hard to say if the conditioned effect we found is beneficial for patients. We did not observe a reduction in blood glucose, which is the primary aim of most diabetes treatments. Conditioning did appear to stabilize blood glucose levels, at least during the test period in men. Instability of plasma glucose levels has been shown to promote microvascular and macrovascular complications such as retinopathy, nephropathy and heart disease (42, 43), and the importance of stabilizing glucose levels is widely discussed in literature (44, 45). Therefore, the effects found in our study may be beneficial for patients, however, it needs to be investigated further.

We have also found that conditioning with intranasal insulin stabilized hunger in healthy participants, partially confirming our study hypothesis. As blood insulin levels rapidly rise after food intake and insulin penetrates the blood brain barrier, it serves as one of the signals to the central nervous system, and particularly the hypothalamus, to stop feeding and decrease hunger (46). Intranasal insulin has been shown to affect hypothalamic neuronal activity (19). Perhaps conditioning with intranasal insulin triggers neuronal activity in the hypothalamus that dampens appetite. However, this effect was found only in healthy controls and not in patients with diabetes type-2. Patients in our sample did not have any increase in hunger during the sessions, even though they had significantly higher baseline hunger than healthy controls. This finding is in keeping with previous research that found that obese patients and patients with diabetes type-2 might be less responsive to the metabolic effects of intranasal insulin (39, 40).

In apparent contrast, no effects of intranasal insulin or conditioning were found on the calories consumed. The total amount of calories eaten during the taste test was very low, possibly because participants knew that the experiment was almost over and they could have a larger meal shortly. For future research, we would propose a more substantial meal, for example, a lunch buffet, to measure food consumption.

No effect of intranasal insulin or insulin conditioning was found on memory. This does not align with several study findings that intranasal insulin administration improves memory in both healthy controls and patients with memory impairments (47, 48). However, most of the studies that found memory-improving effects of intranasal insulin, investigated the effects of long-term treatment of several weeks (49, 50, 51). In our study, we administered 120 units once, which may have been not enough to have an effect on memory. It is worthwhile to investigate whether extending the learning phase of conditioning, and administering higher doses of intranasal insulin, would lead to conditioned memory improvement.

The sex differences found in our study align with previous research findings of the effects of intranasal insulin. Sex differences were found in previous research on the effects of insulin on declarative and working memory and food intake (52, 53), however not all studies replicated these findings (54). The evidence on sex differentiation in intranasal insulin effects is very mixed to this point and appears to be dependent on the timing of administration and health status of participants. Moreover, it remains unknown whether age specific sex differences in the responses to intranasal insulin exist and whether they might have played a role in the findings of the present study.

Several limitations of our study must be mentioned. First, it is important to mention that due to technical issues, we were unable to produce a placebo spray with the same preservative as the insulin spray. Because of this, insulin and placebo sprays had different smells, even though both smells can be described as “medical”. Participants in the conditioned group might have consciously or unconsciously noticed the difference in the way the spray smelled between day 1 and day 2, even though the spray administration were preceded and followed by the administration of a strong smell of aroma oil. However, none of the participants reported noticing the difference, and moreover, when we asked them about the perceived group allocation, the majority of them were not able to correctly tell what spray they received on what day. Despite that, it is important to emphasize that such modification of a part of the conditioned stimulus (that constituted of the rosewood smell, smell of the spray and the context of spray administration), might possibly have led to a diminished conditioned response. At the same time, we do not expect that this change would completely have blocked the conditioning, as it has been shown that when the CS presented during the evocation phase is slightly different than the CS presented during the acquisition phase, the conditioned effect remains present (55). Secondly, the findings related to sex differences were done in exploratory analyses as we had no directional hypothesis regarding sex effects. However, considering sex differences found in previous research, we recruited similar numbers of men and women in each of the experimental groups. A previous experiment documenting a metabolic effect of insulin conditioning (7) studied only men, which matches our findings, showing (albeit opposite) conditioned effects on glucose levels in men only. The impact of sex on the metabolic effects of insulin conditioning needs to be confirmed in a study that is specifically powered to detect sex differences. Third, the men-women ratio in our study is not entirely equal: due to practical issues and the constraints the COVID-19 pandemic posed, we had to deviate slightly from an equal balance. Finally, the results found in our study are not necessarily generalizable to patients with more severe diabetes type-2. We intentionally included only patients with milder disease, who were treated either with behavioral interventions or metformin, and not patients who received insulin injections. Patients with severe insulin resistance or a significant loss of beta-cells might be less responsive to conditioning manipulations. Finally, it is important to mention that the conditioned stimulus, rosewood oil, might have had certain physiological and psychological effects. Rosewood oil is rich in linalool that has antioxidant and anxiolytic effects (56). However, these effects were found only in studies with administrations of larger doses of linalool (56), and there is not enough evidence that smelling oil for several minutes as was done in our study, is enough to produce any significant effects. Moreover, as the CS was given in both experimental and control groups, its effects would not be reflected in the between-group comparison.

Our study has several important implications. We demonstrated that conditioning with intranasal insulin reduces hunger in healthy participants. Hunger can be a problem not only for patients with diabetes type-2 but for populations who needs to follow a diet for other health reasons. Applying intranasal insulin conditioning can help these groups of people. Importantly, we provided further evidence that glucose responses can be conditioned, not only in healthy controls but also in male patients with diabetes type-2. However, our results indicate that sex and disease specific effects might play a role in endocrine conditioning and better understanding of these effects is needed.

**Acknowledgements**

We would like to thank Ursula Stockhorst for the input on parts of the study protocol. We also thank Monique Smeets and Ilja Croijmans for their help with preparation of the fragrant pens. Participant recruitment was accomplished through Hersenonderzoek.nl, a Dutch online registry that facilitates participant recruitment for neuroscience studies (www.hersenonderzoek.nl). Hersenonderzoek.nl is funded by ZonMw-Memorabel (project number 73305095003), a project in the context of the Dutch Deltaplan Dementie, Gieskes-Strijbis Foundation, the Alzheimer’s Society in the Netherlands and Brain Foundation Netherlands.

**Author Contributions**

All authors were responsible for drafting the article and revising it critically for important intellectual content. All authors approved the version to be published.

**Declaration of interests**

None

**References**

1. Colloca L, Miller FG. Role of expectations in health. Current opinion in psychiatry. 2011 Mar 1;24(2):149-55.

2. Skvortsova, A., Veldhuijzen, D.S., Kloosterman, I.E.M., Meijer, O.C., van Middendorp, H., Pacheco-Lopez, G., Evers, A.W.M., 2019a. Conditioned hormonal responses: A systematic review in animals and humans. Front Neuroendocrinol 52, 206–218.

3. Tekampe, J., van Middendorp, H., Meeuwis, S.H., van Leusden, J.W.R., Pacheco-López, G., Hermus, A.R.M.M., Evers, A.W.M., 2017. Conditioning Immune and Endocrine Parameters in Humans: A Systematic Review. Psychother Psychosom 86, 99–107. <https://doi.org/10.1159/000449470>.

4. Doering, B.K., Rief, W., 2012. Utilizing placebo mechanisms for dose reduction in pharmacotherapy. Trends in pharmacological sciences 33, 165–172.

5. Tekampe, J., van Middendorp, H., Sweep, F.C.G.J., Roerink, S.H.P.P., Hermus, A.R.M.M., Evers, A.W.M., 2018. Human Pharmacological Conditioning of the Immune and Endocrine System: Challenges and Opportunities. Int. Rev. Neurobiol. 138, 61–80. <https://doi.org/10.1016/bs.irn.2018.01.002>

6. Kirchhof, J., Petrakova, L., Brinkhoff, A., Benson, S., Schmidt, J., Unteroberdörster, M., et al, 2018. Learned immunosuppressive placebo responses in renal transplant patients. Proc. Natl. Acad. Sci. U.S.A. 115, 4223–4227. https://doi.org/10.1073/pnas.1720548115

7. Stockhorst, U., De Fries, D., Steingrueber, H.-J., Scherbaum, W.A., 2011. Unconditioned and conditioned effects of intranasally administered insulin vs placebo in healthy men: a randomised controlled trial. Diabetologia 54, 1502–1506.

8. Stockhorst, U., Gritzmann, E., Klopp, K., Schottenfeld-Naor, Y., Hubinger, A., Berresheim, H.-W., Steingruber, H.-J., Gries, F.A., 1999. Classical conditioning of insulin effects in healthy humans. Psychosomatic medicine 61, 424–435.

9. Stockhorst, Ursula, Huenig, A., Ziegler, D., Scherbaum, W.A., 2011. Unconditioned and conditioned effects of intravenous insulin and glucose on heart rate variability in healthy men. Physiology & behavior 103, 31–38.

10. Stockhorst, U., Mahl, N., Krueger, M., Huenig, A., Schottenfeld-Naor, Y., Huebinger, A., et al, 2004. Classical conditioning and conditionability of insulin and glucose effects in healthy humans. Physiology & behavior 81, 375–388.

11. Stockhorst, U., Steingrüber, H.-J., Scherbaum, W.A., 2000. Classically conditioned responses following repeated insulin and glucose administration in humans. Behavioural brain research 110, 143–159.

12. Skvortsova, A., Veldhuijzen, D.S., Kloosterman, I.E., Pacheco-López, G., Evers, A.W., 2021. Food anticipatory hormonal responses: A systematic review of animal and human studies. Neuroscience & Biobehavioral Reviews 126, 447–464.

13. Smeets, P.A., Erkner, A., De Graaf, C., 2010. Cephalic phase responses and appetite. Nutrition Reviews 68, 643–655.

14. Detke, M.J., Brandon, S.E., Weingarten, H.P., Rodin, J., Wagner, A.R., 1989. Modulation of behavioral and insulin responses by contextual stimuli paired with food. Physiology & behavior 45, 845–851.

15. Roozendaal, B., Oldenburger, W.P., Strubbe, J.H., Koolhaas, J.M., Bohus, B., 1990. The central amygdala is involved in the conditioned but not in the meal-induced cephalic insulin response in the rat. Neuroscience letters 116, 210–215.

16. Surwit, R.S., McCubbin, J.A., Livingston, E.G., Feinglos, M.N., 1985. Classically conditioned hyperglycemia in the obese mouse. Psychosomatic medicine 47, 565–568.

17. Born, J., Lange, T., Kern, W., McGregor, G.P., Bickel, U., Fehm, H.L., 2002. Sniffing neuropeptides: a transnasal approach to the human brain. Nature neuroscience 5, 514.

18. Vidarsdottir, S., Smeets, P.A., Eichelsheim, D.L., van Osch, M.J., Viergever, M.A., Romijn, J.A., van der Grond, J., Pijl, H., 2007. Glucose ingestion fails to inhibit hypothalamic neuronal activity in patients with type 2 diabetes. Diabetes 56, 2547–2550.

19. van Opstal, A.M., Akintola, A.A., van der Elst, M., Westendorp, R.G., Pijl, H., van Heemst, D., van der Grond, J., 2017. Effects of intranasal insulin application on the hypothalamic BOLD response to glucose ingestion. Scientific reports 7, 13327.

20. Hallschmid, M., Higgs, S., Thienel, M., Ott, V., Lehnert, H., 2012. Postprandial administration of intranasal insulin intensifies satiety and reduces intake of palatable snacks in women. Diabetes 61, 782–789. https://doi.org/10.2337/db11-1390

21. Jauch-Chara, K., Friedrich, A., Rezmer, M., Melchert, U.H., Scholand-Engler, H.G., Hallschmid, M., Oltmanns, K.M., 2012. Intranasal insulin suppresses food intake via enhancement of brain energy levels in humans. Diabetes 61, 2261–2268.

22. Novak, V., Milberg, W., Hao, Y., Munshi, M., Novak, P., Galica, A., et al, 2014. Enhancement of vasoreactivity and cognition by intranasal insulin in type 2 diabetes. Diabetes care 37, 751–759.

23. Ott, V., Benedict, C., Schultes, B., Born, J., Hallschmid, M., 2012. Intranasal administration of insulin to the brain impacts cognitive function and peripheral metabolism. Diabetes, obesity and metabolism 14, 214–221.

24. Lillioja, S., Mott, D.M., Spraul, M., Ferraro, R., Foley, J.E., Ravussin, E., et al., 1993. Insulin resistance and insulin secretory dysfunction as precursors of non-insulin-dependent diabetes mellitus: prospective studies of Pima Indians. New England journal of medicine, 329(27), pp.1988-1992.

25. Avignon A, Radauceanu A, Monnier L. Nonfasting plasma glucose is a better marker of diabetic control than fasting plasma glucose in type 2 diabetes. Diabetes care. 1997 Dec 1;20(12):1822-6.

26. Berger, B., Stenström, G. and Sundkvist, G., 2000. Random C-peptide in the classification of diabetes. Scandinavian journal of clinical and laboratory investigation, 60(8), pp.687-693.

27. Weyer, C., Hanson, R.L., Tataranni, P.A., Bogardus, C. and Pratley, R.E., 2000. A high fasting plasma insulin concentration predicts type 2 diabetes independent of insulin resistance: evidence for a pathogenic role of relative hyperinsulinemia. Diabetes, 49(12), pp.2094-2101.

28. Skvortsova, A., Veldhuijzen, D.S., Pacheco-Lopez, G., Bakermans-Kranenburg, M., van IJzendoorn, M., Smeets, M.A., et al., 2019b. Placebo effects in the neuroendocrine system: conditioning of the oxytocin responses. Psychosomatic medicine.

29. Zech, H.G., Rotteveel, M., van Dijk, W.W., van Dillen, L.F., 2020. A mobile approach-avoidance task. Behavior Research Methods 52, 2085–2097.

30. Blechert, J., Meule, A., Busch, N.A., Ohla, K., 2014. Food-pics: an image database for experimental research on eating and appetite. Frontiers in psychology 5, 617.

31. Schakel, L., Veldhuijzen, D.S., Middendorp, H. van, Dessel, P.V., Houwer, J.D., Bidarra, R., Evers, A.W., 2018. The effects of a gamified approach avoidance training and verbal suggestions on food outcomes. PloS one 13, e0201309.

32. van den Akker, K., Schyns, G., Jansen, A., 2016. Enhancing inhibitory learning to reduce overeating: Design and rationale of a cue exposure therapy trial in overweight and obese women. Contemporary clinical trials 49, 85–91.

33. Lezak, M.D., Howieson, D.B., Loring, D.W., Fischer, J.S., 2004. Neuropsychological assessment. Oxford University Press, USA.

34. Cohen, J., 1988. Statistical Power Analysis for the Behavioral Sciences. Hillsdle. Erlbaum. Conner, BE (1988). The Box in the Barn. Columbus.

35. Tekampe, J., van Middendorp, H., Biermasz, N.R., Sweep, F.C., Meijer, O.C., Pelsma, I.C., et al, 2021. Conditioning cortisol in healthy young women–A randomized controlled trial. Psychoneuroendocrinology, 124, p.105081.

36. Benedict, C., Brede, S., Schiöth, H.B., Lehnert, H., Schultes, B., Born, J., Hallschmid, M., 2011. Intranasal insulin enhances postprandial thermogenesis and lowers postprandial serum insulin levels in healthy men. Diabetes 60, 114–118.

37. Dash, S., Xiao, C., Morgantini, C., Koulajian, K., Lewis, G.F., 2015. Intranasal insulin suppresses endogenous glucose production in humans compared with placebo in the presence of similar venous insulin concentrations. Diabetes 64, 766–774.

38. Leary, A.C., Stote, R.M., Breedt, H.J., O’brien, J., Buckley, B., 2005. Pharmacokinetics and pharmacodynamics of intranasal insulin administered to healthy subjects in escalating doses. Diabetes technology & therapeutics 7, 124–130.

39. Xiao, C., Dash, S., Stahel, P., Lewis, G.F., 2018. Effects of intranasal insulin on endogenous glucose production in insulin-resistant men. Diabetes, Obesity and Metabolism 20, 1751–1754.

40. Gancheva, S., Koliaki, C., Bierwagen, A., Nowotny, P., Heni, M., Fritsche, A., et al, 2015. Effects of intranasal insulin on hepatic fat accumulation and energy metabolism in humans. Diabetes 64, 1966–1975.

41. Roberts, S.B., Rosenberg, I., 2006. Nutrition and aging: changes in the regulation of energy metabolism with aging. Physiological reviews 86, 651–667.

42. Diabetes Control and Complications Trial Research Group, 1995. The relationship of glycemic exposure (HbA1c) to the risk of development and progression of retinopathy in the diabetes control and complications trial. Diabetes, 44(8), pp.968-983.

43. Klein, R., 1995. Hyperglycemia and microvascular and macrovascular disease in diabetes. Diabetes Care 18, 258–268. <https://doi.org/10.2337/diacare.18.2.258>

44. Gómez, A.M., Muñoz, O.M., Marin, A., Fonseca, M.C., Rondon, M., Robledo Gómez, M.A., et al, 2018. Different indexes of glycemic variability as identifiers of patients with risk of hypoglycemia in type 2 diabetes mellitus. Journal of diabetes science and technology 12, 1007–1015.

45. Monnier, L., Colette, C., 2008. Glycemic variability: should we and can we prevent it? Diabetes care 31, S150–S154.

46. Hallschmid, M., Schultes, B., Marshall, L., Mölle, M., Kern, W., Bredthauer, J., et al, 2004. Transcortical direct current potential shift reflects immediate signaling of systemic insulin to the human brain. Diabetes 53, 2202–2208.

47. Benedict, C., Frey II, W.H., Schiöth, H.B., Schultes, B., Born, J., Hallschmid, M., 2011b. Intranasal insulin as a therapeutic option in the treatment of cognitive impairments. Experimental gerontology 46, 112–115.

48. Hallschmid, M., 2021. Intranasal insulin. Journal of Neuroendocrinology 33, e12934.

49. Benedict, C., Hallschmid, M., Hatke, A., Schultes, B., Fehm, H.L., Born, J., Kern, W., 2004. Intranasal insulin improves memory in humans. Psychoneuroendocrinology 29, 1326–1334.

50. Benedict, C., Hallschmid, M., Schmitz, K., Schultes, B., Ratter, F., Fehm, H.L., et al, 2007. Intranasal insulin improves memory in humans: superiority of insulin aspart. Neuropsychopharmacology 32, 239–243.

51. Hallschmid, M., Benedict, C., Schultes, B., Born, J., Kern, W., 2008. Obese men respond to cognitive but not to catabolic brain insulin signaling. International journal of obesity 32, 275–282.

52. Benedict, C., Hallschmid, M., Schultes, B., Born, J., Kern, W., 2007b. Intranasal insulin to improve memory function in humans. Neuroendocrinology 86, 136–142.

53. Claxton, A., Baker, L.D., Wilkinson, C.W., Trittschuh, E.H., Chapman, D., Watson, G., et al., 2013. Sex and ApoE genotype differences in treatment response to two doses of intranasal insulin in adults with mild cognitive impairment or Alzheimer’s disease. Journal of Alzheimer’s Disease 35, 789–797.

54. Santiago, J.C., Hallschmid, M., 2017. Central nervous insulin administration before nocturnal sleep decreases breakfast intake in healthy young and elderly subjects. Frontiers in neuroscience 11, 54.

55. Kampermann, L., Tinnermann, A. and Büchel, C., 2021. Generalization of placebo pain relief. Pain, 162(6), pp.1781-1789.

56. Aprotosoaie, A.C., Hăncianu, M., Costache, I.I. and Miron, A., 2014. Linalool: a review on a key odorant molecule with valuable biological properties. Flavour and fragrance journal, 29(4), pp.193-219.

**Table 1**. Baseline characteristics and taste test, approach-avoidance task and memory scores with means, standard errors in parentheses and number of observations in square brackets across groups and study conditions.

|  | Conditioned group | | Control group | | |
| --- | --- | --- | --- | --- | --- |
|  | Patients | Healthy controls | Patients | Healthy controls | |
| Age (years) | 68.31 (2.37) [16] | 67.69 (2.37) [16] | 68.20 (2.44) [16] | 67.81(5.5) [16] | |
| Body mass index | 29.77 (0.84) [16] | 25.08 (0.84) [16] | 27.77 (0.87) [16] | 25.92 (0.84) [16] | |
| Baseline insulin, day 1(mU/L) | 14.59 (2.17) [16] | 9.01 (2.17) [16] | 12.69 (2.17) [16] | 12.07 (2.17) [16] | |
| Baseline glucose, day 1 (mmol/L) | 8.49 (0.28) [16] | 5.35 (0.28) [16] | 8.36 (0.28) [16] | 5.43 (0.28) [16] | |
| Baseline c-peptide, day 1 (mmol/L) | 1.12 (0.10) [16] | 0.74 (0.10) [16] | 1.14 (0.10) [16] | 0.90 (0.10) [16] | |
| Baseline hunger, day 1 | 4.5 (0.64) [16] | 2.09 (0.64) [16] | 5.2 (0.66) [16] | 2.69 (0.64) [16] | |
| Baseline insulin, day 2 (mU/L) | 12.8 (2.10) [16] | 9.42 (2.10) [16] | 10.67 (2.17) [16] | 13.11 (2.10) [16] | |
| Baseline glucose, day 2 (mmol/L) | 8.34 (0.30) [16] | 5.28 (0.30) [16] | 8.18 (0.31) [16] | 5.41 (0.30) [16] | |
| Baseline c-peptide, day 2 (mmol/L) | 1.08 (0.10) [16] | 0.77 (0.10) [16] | 1.07 (0.10) [16] | 0.94 (0.10) [16] | |
| Baseline hunger, day 2 | 3.91 (0.60) [16] | 2.59 (0.60) [16] | 4.73 (0.62) [16] | 4.19 (0.60) [16] | |
| Taste test day 1 (kcal) | 74.99 (15.66) [16] | 74.23 (20.56) [16] | 142.26 (44.79) [16] | | 71.59 (17.49) [16] |
| Taste test day 2 (kcal) | 78.77 (19.72) [16] | 89.62 (23.7) [16] | 127.45 (37.58) [16] | | 62.47 (13.95) [16] |
| Approach to food reaction time day 1 (seconds)* | 0.17 (0.23) [14] | 0.14 (0.25) [14] | 0.17 (0.31) [15] | | 0.25 (0.29) [15] |
| Approach to food reaction time day 2 (seconds)* | 0.21 (0.22) [14] | 0.25 (0.25) [14] | 0.28 (0.24) [15] | | 0.31 (0.21) [15] |
| Approach to food force day 1 (meters/seconds^2)* | 0.43 (5.96) [14] | 3.93 (6.27) [14] | -1.42 (6.75) [15] | | 0.91(7.41) [15] |
| Approach to food force day 2 (meters/seconds^2)* | 2.29 (6.33) [14] | 1.98 (5.95) [14] | 0.56 (7.50) [15] | | -0.89 (8.48) [15] |
| Immediate recall day 1 | 44.60 (2.74) [10] | 43.00 (2.50) [12] | 40.89 (2.89) [10] | | 40.42 (2.50) [13] |
| Immediate recall day 2 | 45.20 (2.80) [11] | 46.25 (2.55) [12] | 40.78 (2.95) [10] | | 41.58 (2.55) [12] |
| Learning day 1 | 5.30 (0.56) [10] | 5.33 (0.51) [12] | 5.67 (0.59) [10] | | 4.67 (0.51) [13] |
| Learning day 2 | 5.90 (0.84) [11] | 5.75 (0.76) [12] | 4.67 (0.88) [10] | | 5.33 (0.76) [12] |
| Percent forgetting day 1 | 0.21 (0.07) [10] | 0.27 (0.06) [12] | 0.269 (0.07) [10] | | 0.164 (0.06) [13] |
| Percent forgetting day 2 | 0.24 (0.07) [11] | 0.36 (0.06) [12] | 0.25 (0.07) [10] | | 0.22 (0.06) [12] |

*difference between pull and push conditions

**Table 2.** The factorial ANOVAs comparing groups and conditions on memory scores

| Variable | Factor | F | p | η_p_^2^ |
| --- | --- | --- | --- | --- |
| Immediate recall day 1 | Condition (conditioned versus control) | 0.37 | .544 | .009 |
|  | Group (patients versus healthy controls) | 0.02 | .885 | .001 |
|  | Condition*Group | 0.003 | .960 | <.001 |
| Immediate recall day 2 | Condition (conditioned versus control) | 1.48 | .231 | .035 |
|  | Group (patients versus healthy controls) | 0.45 | .505 | .011 |
|  | Condition*Group | 0.22 | .646 | .005 |
| Learning day 1 | Condition (conditioned versus control) | 0.04 | .853 | .001 |
|  | Group (patients versus healthy controls) | 0.62 | .434 | .015 |
|  | Condition*Group | 1.94 | .171 | .045 |
| Learning day 2 | Condition (conditioned versus control) | 0.45 | .508 | .011 |
|  | Group (patients versus healthy controls) | 0.16 | .691 | .004 |
|  | Condition*Group | 0.02 | .886 | .001 |
| Percent forgetting day 1 | Condition (conditioned versus control) | 0.19 | .666 | .005 |
|  | Group (patients versus healthy controls) | 0.19 | .663 | .005 |
|  | Condition*Group | 1.78 | .189 | .043 |
| Percent forgetting day 2 | Condition (conditioned versus control) | 0.88 | .354 | .022 |
|  | Group (patients versus healthy controls) | 0.43 | .515 | .011 |
|  | Condition*Group | 1.15 | .290 | .029 |

**FIGURE CAPTIONS**

**Figure 1.** The mean changes of glucose levels from baseline with standard errors.

**Figure 2.** The mean changes of hunger from baseline with standard errors.
