# Supplementary figures and images for: Influencing the Insulin System by Placebo Effects in Patients With Diabetes Type 2 and Healthy Controls: A Randomized Controlled Trial
Source: Psychosom Med. 2023 Jun 23;85(6):551–60. doi: 10.1097/PSY.0000000000001216 (PMC10332649; doi:10.1097/PSY.0000000000001216)

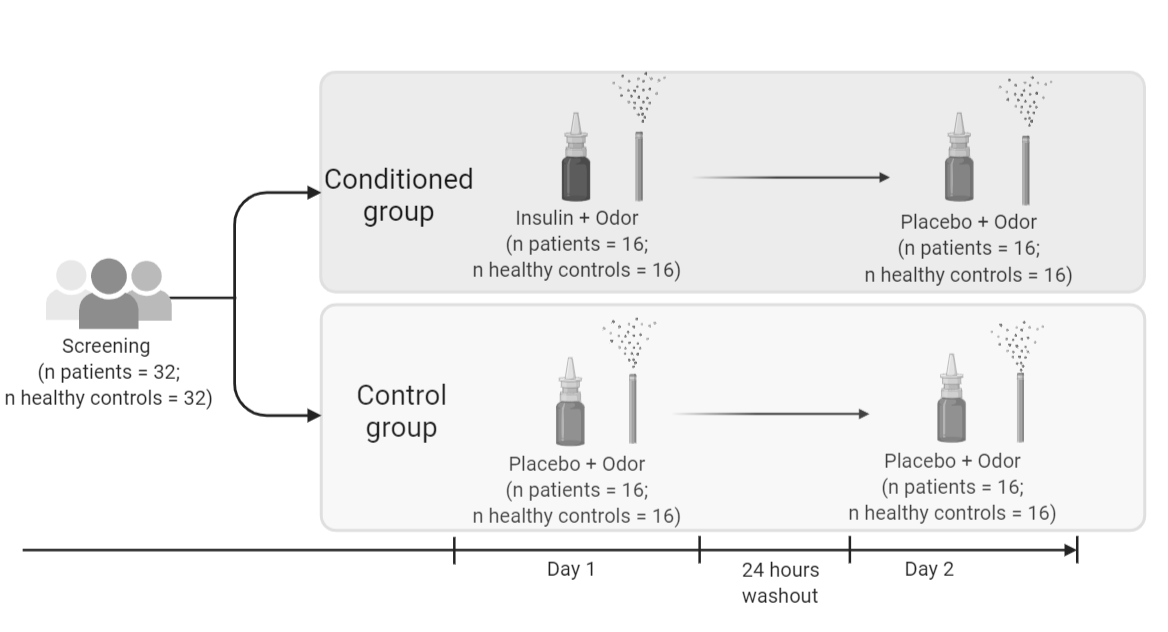

Supplement: Supplementary file 3 [file psymed-85-551-s003.png]

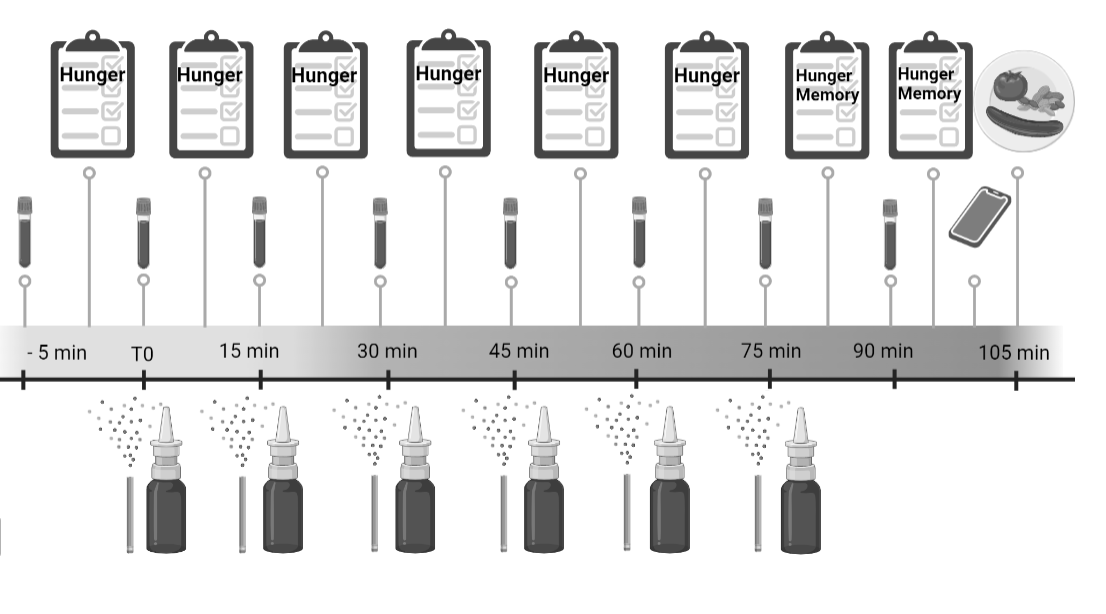

Supplement: Supplementary file 4 [file psymed-85-551-s004.png]

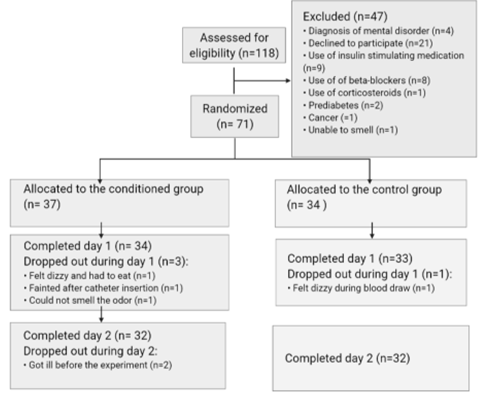

Supplement: Supplementary file 5 [file psymed-85-551-s005.tif]

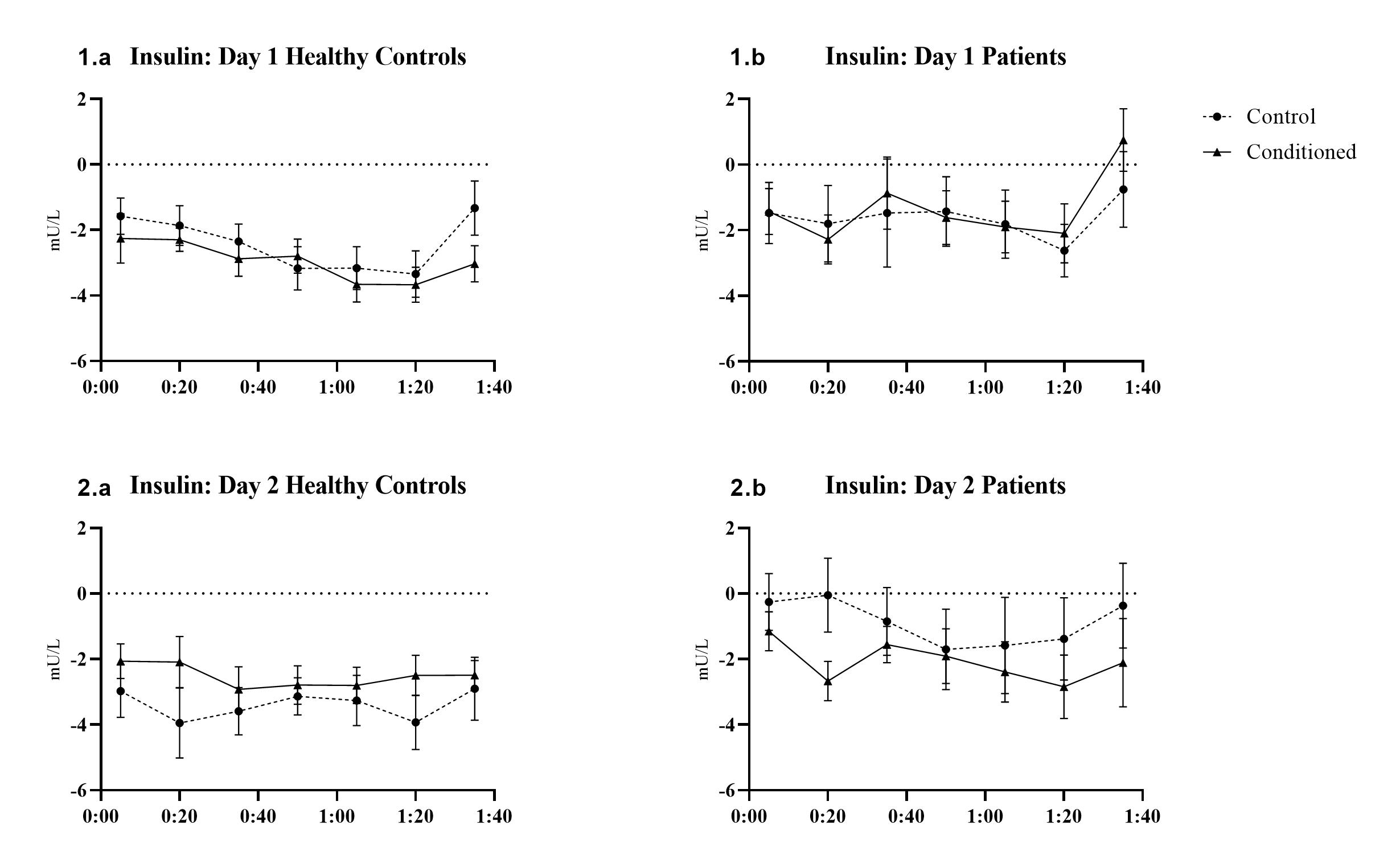

Supplement: Supplementary file 6 [file psymed-85-551-s006.jpg]

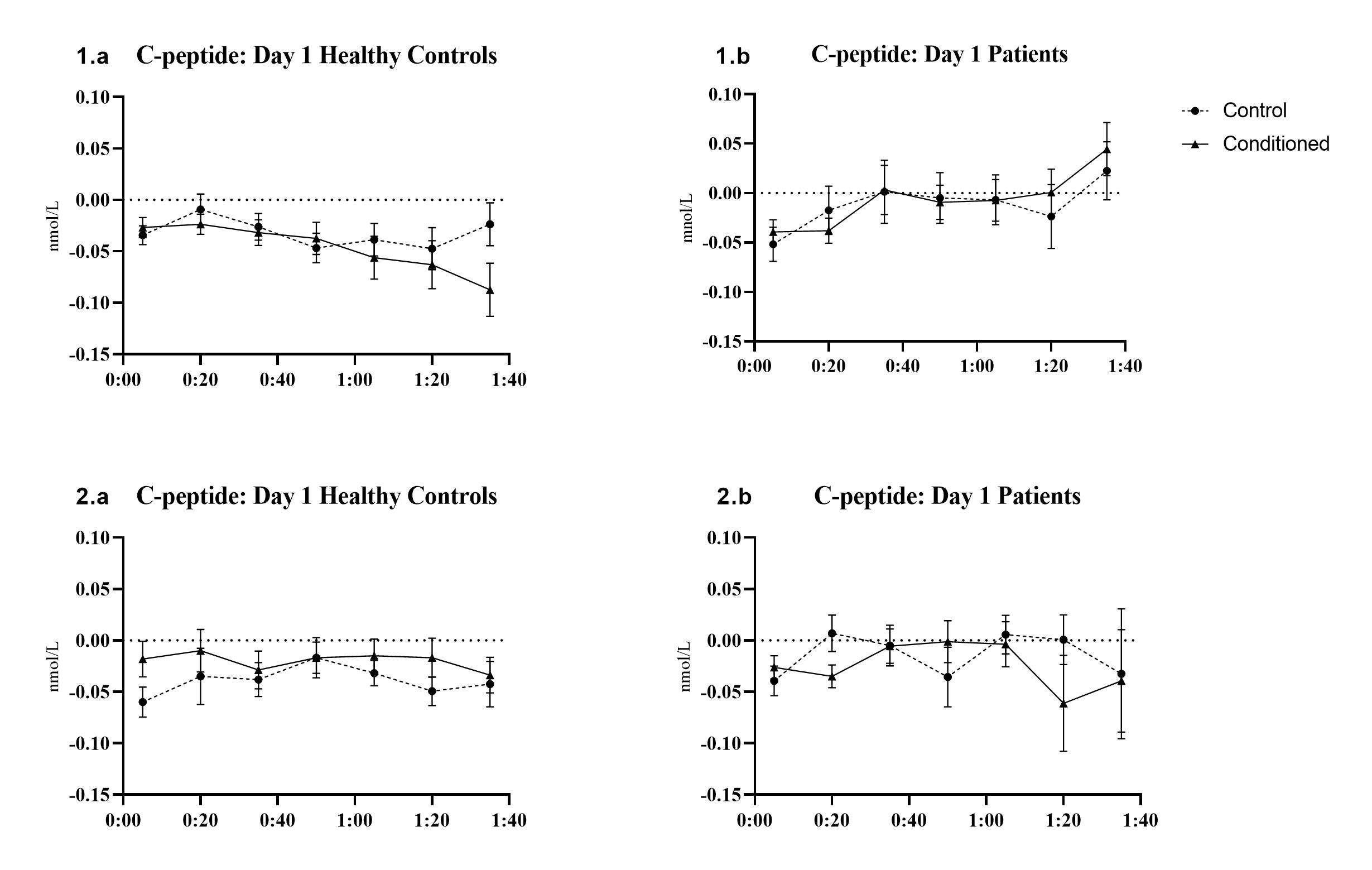

Supplement: Supplementary file 7 [file psymed-85-551-s007.jpg]
